# Road urban planning sustainability based on remote sensing and satellite dataset: A review

**DOI:** 10.1016/j.heliyon.2024.e39567

**Published:** 2024-10-18

**Authors:** Khalid Hardan Mhana, Shuhairy Bin Norhisham, Herda Yati Binti Katman, Zaher Mundher Yaseen

**Affiliations:** a Institute of Energy Infrastructure (IEI) and Department of Civil Engineering, College of Engineering, Universiti Tenaga Nasional (UNITEN), Putrajaya Campus, Jalan IKRAM-UNITEN, 43000, Kajang, Selangor, Malaysia; b Civil Engineering Department, College of Engineering, University of Anbar, Iraq; c Civil and Environmental Engineering Department, King Fahd University of Petroleum & Minerals, Dhahran, 31261, Saudi Arabia

**Keywords:** Road network, Infrastructural development, Environmental impact, Urban development, Literature review

## Abstract

Infrastructural development and urbanization effects have been investigated over the past decades with novel approaches and adaptation strategies. Road network expansions are more useful for the socio-economic development from urban to rural areas where 75 % of the passenger, and goods transportation sectors are influenced by the road. Road infrastructure and urbanization are perpendicular to each other, and this research investigation indicates that the novel approaches and adaptation strategies for road infrastructure and urbanization effects. This study evaluated the trend in the road network and urbanization-related literature from 2010 to 2022 with some measurable keywords. Around 370 pieces of research literature are analysis and around 85 research evaluations for the road network and urbanization-related Land use and land cover (LULC) studies while numerous road network analysis approaches and LULC-related investigations are evaluated in this research. Three major parts road network analysis-related approaches, LULC, and urbanization-related approaches related to road network expansion and urbanization, were investigated. In this work, many research publications' approaches to LULC simulation, kernel density, shortage distance, and picture classification are discussed and assessed. The survey is more valuable for urban planners, future disaster management teams, and administrators to implement the shortage distance analysis, reduction of road accidents, and urbanization effects on the environment.

## Abbreviations

ANN: Artificial Neural Network
AI: Artificial Intelligence
AHP: Analytical Hierarchy Process
ASTER: Advanced Thermal Emission and Reflection Radiometer
AADT: Annual Average Daily Traffic
ATM: Automated teller machine
CA: Cellular Automata
CLIQUE: CLustering In QUEst
CNN: Conventional Neural Netwrok
CURE: Clustering Using REpresentatives
DEM: Digital Elevation Model
DBSCAN: Density-Based Spatial Clustering of Applications with Noise
DENCLUE: DENsity-based CLUstEring
DEA: Data Envelopment Analysis
DRAM: Disaggregate Residential Allocation Model
ETM+: Enhance Thematic Mapper Plus
EMPAL: Employment Allocation Model
FST: Fuzzy set theory
FFNN: Feed forward neural networks
FR: Frequency Ratio
GIS: Geographic Information System
GML: Gaussian Maximum Likelihood
ISRO: Indian Space Research Organisation
ITLUP: Integrated Transportation and Land Use Package
KDE: Kernel Density Estimation
LULC: Land Use and Land Cover
LSTM: Long-short Term Memory
LR: Linear Regression
LCM: Land change modeler
ML: Machine Learning
MLPNN: Multiple-layer Perceptron Neural Network
MODIS: Moderate Resolution Imaging Spectroradiometer
MCDA: Multi-criteria Decision Analysis
MOLUSCE: Modules for Land Use Change Evaluation
MSS: Multi-Spectral Scanner
MCE: Multi-criteria Evaluation
MD: Mahalanobis Distance
NASA: National Aeronautics and Space Administration
OLI/TIRS: Operational Land Imager/Thermal Infrared Sensor
PCA: Principal Component Analysis
PCR: Principal Component Regression
RTA: Road Traffic Accident
RF: Random Forest
R: Coefficient of Correlation
RBFNN: Radial Base Function Neural Network
RMSE: Root Mean Square Errors
MAD: Mean Absolute Deviation
MAE: Mean Absolute Errors
SDG: Sustainable Development Goals
SRTM: Shuttle Radar Topography Mission
SIC: Standard Industrial Classification
SVM: Support Vector Machine
SPI: Standard Precipitation Index
SMA: Spectral Mixture Analysis
STING: STatistical INformation Grid
SD: Standard Deviation
SI: Severity Index
TM: Thematic Mapper
UHI: Urban Heat Island
USGS: United States Geological Survey
UGM: Urban Growth Model
WoE: Weights of Evidence

## 1 Introduction

### 1.1 Research background

**Fig. 1:**
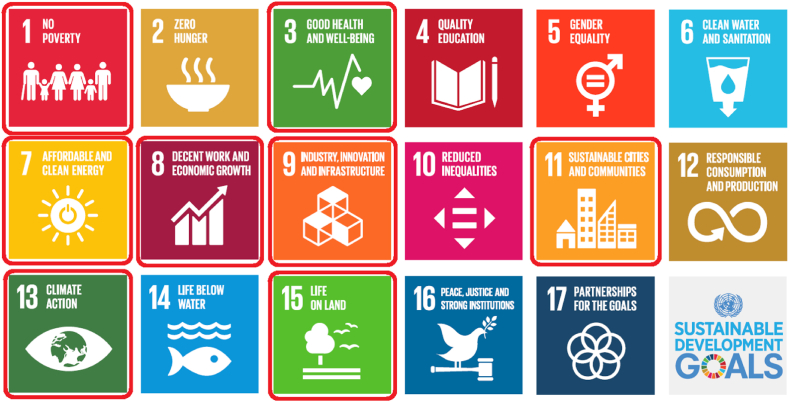
UN Sustainable Development Goals (https://sdgs.un.org/goals). The study mainly focused on SDGs 1, 3, 7, 8, 9, 11, 13, and 15.

The network system refers to the infrastructural development and transportation variability which is a linkage to the connectivity of the different lands [[1], [2], [3], [4]]. The connection points are also known as vertices or nodes, which characterize some particular stations, destinations, emergency points, or junctions [5,6]. Infrastructural development and Urban sprawl create major impacts on mobility, land use, and energy sources [[7], [8], [9]]. The road and related utilities are gradually increased because of urbanization and anthropogenic density. Many areas are converted into roads where densely residential areas are located. Furthermore, fringe areas are also developing new roads for transportation purposes and connecting to other parts of the country or territory [10]. Various transportation systems are noticed but around 75 % of transportation is dependent on road networks. The urban and rural areas are connected with road networks, also the agricultural sector and industrial sectors are using the road network for production transformation, market availability, and economic development, therefore road networks play a vital role in urban amenities and rural development [[11], [12], [13], [14], [15]]. The road set-up positively increases the economic condition because this way of transport has low cost but the road development also triggers environmental degradation like green space dynamics, urban expansion, heat variation, CO_2_ emission, air pollution, and health issues [[16], [17], [18]]. Road development can also be used for modernization and labor transfer from rural or fringe areas (agricultural-based environment) to urban areas (industrial-based environment), where fossil fuels are gradually increasing and create air pollution, heat islands, and health issues [19,20]. Therefore, road infrastructures play a significant role in urban development and also environmental degradation in the earth's crust. Some transportation systems depend on weather conditions; even those systems can be delayed or canceled due to extreme weather conditions. Therefore, road network development is essential for any region or country's economic, social, and cultural development. Climate change may also be a catalyst for issues with the road network, encouraging consideration of improved road network design and traffic safety. Road-related investigations like road accident-prone area identification, road safety, traffic control, suitable highway selection, and other urban amenities area identification are more important for road development [5,[21], [22], [23], [24]]. Furthermore, road infrastructure can also trigger factors or heat waves [25,26], green space dynamics, soil moisture loss because of high industrial works, LULC alteration, air pollution increase, and many other environmental and health issues. The coming decades will see urbanization as a defining phenomenon, and it already is. Thus, a major factor influencing our total success will be how we handle our urbanization. In urban areas, collaborating with local stakeholders, the SDGs may be most effectively localized. The New Urban Agenda is an essential instrument for speeding up the achievement of the SDGs since it provides direction on development, financing, planning, design, governance, and management to handle the advantages and drawbacks of urbanization (Fig. 1). Sustainable urbanization is a game-changer for accomplishing Agenda 2030. The Sustainable Development Goals are an all-encompassing set of goals and ambitions. The realization of the aims under SDG 11, which are to “make cities inclusive, safe, resilient, and sustainable,” has a revolutionary impact on the achievement of targets under subsequent goals. Achieving good urbanization requires achieving several “urban-critical” targets that are included under other goals.

### 1.2 Research significant

Road infrastructure and environmental issues-related review papers are limited therefore a strong review article can help the researchers, scientists, and management team for better adaptation strategies implementation in a particular area. The road network is used for goods and product services from one plane to another place and this system has more economic value [27]. Around 75 % of passengers and goods are transported to other parts of Sub-Sahara Africa [28]. This region has road and related infrastructural development strategies where 50 % cost is used for road network development, cities, and potential social-economic or physical development [29,30]. Kenya and Ghana have some road network-related strategies that progressively increase with the social and spatial heterogeneity in the peri-urban areas [31]. Furthermore, road traffic accidents (RTA) were the maximum concerning factor in recent eras where urbanization has increased but proper planning of road networks is destroying the planning and increasing road accidents [32].

Previous studies indicate that road accident analysis is riskier but still, people use this transportation. The GIS-based application can help with decision-making with adaptation development in a spatial investigation and short-term periods [33,34]. There are some limitations of road networks and LULC-related information and the environmental impact on the earth's surface. Road density analysis [1,35], traffic volume estimation [36,37], accident-prone area analysis [38], shortage distance, suitable sites, and many other land use phenomena are associated with the use of land that's why the road network analysis or review-related applications are interlinked with the LULC. The road network expansion can also decrease the green space, and agricultural lands, and increase air pollution and health issues but fast transport, low cost, and huge passenger transportation also benefit [[39], [40], [41]]. Hotspot area is applied for the high-risk zone analysis [32,42] and kernel density is estimated for the road density analysis because high-density areas have more heat variation observed and densely built-up lands are also identified in the region [35,43,44]. Some techniques are applied for LULC classification like supervised classification, unsupervised classification, random forest, artificial neural network, deep learning, and object-based classification [45,46]. Different satellite images are also used for image classification like Landsat, Sentinel-1/2, LISS-III, MODIS, and hyper-spectral datasets [[47], [48], [49]]. The urban expansion can also be predicted or simulated using different techniques [[50], [51], [52]]. From the 1970s Landsat datasets were applied for land surface change analysis and predicting the LULC. Spectral Mixture Analysis (SMA), Pixel-based approaches, object-based approaches, knowledge-based approaches, and other methods are mentioned in the review article where this was focused on Landsat datasets like Operational Land Imager/Thermal Infrared Sensor (OLI/TIRS), Enhance Thematic Mapper Plus (ETM+), Thematic Mapper (TM), and Multi-Spectral Scanner (MSS) [53]. This review article applies different approaches for image classification based on the Landsat datasets. Studies that conducted reviews of the literature revealed that the World War II Road linearity. In addition, planners, and researchers can understand that road construction and management of road safety are essential for future urban management [54]. Some machine learning methods are applied for Landsat data classification like Artificial Neural Network (ANN), Random Forest (RM), Spectral Angle mapper, Support Vector Machine (SVM), and Mahalanobis Distance (MD) were applied for image classification [55].

Some researchers have applied road-LULC-related applications like MEPLAN, STASA, TRANUS, and UrbanSim for the society and economic aspect, there are some other methods like Cellular Automata (CA), Analytical Hierarchy Process (AHP), Markov-CA model, SLEUTH model, Fuzzy set theory (FST), and agent-based model reviewed for the urban management [56]. A review paper is an analysis of the CA model application in the urban application [57], wherever numerous categories of research papers were mentioned with the application. Additionally, new validation approaches expansion based on the acknowledgment of urban design and the examination of the CA's potential combination with additional conventional urban and geographical theories would be included in the review research in the chosen method. This would provide the urban CA with an improved industrialized theoretical circumstantial framework [57]. A group of researchers ANN behalf of the analysis of road safety where the coefficient of correlation (R), mean absolute percentage errors (MAPE), root mean square errors (RMSE), mean absolute deviation (MAD), mean absolute errors (MAE), and coefficient (R^2^), etc. [4]. Our review is an appropriate analysis of the related 85 articles published on road safety and LULC-related issues in recent periods which reviewed the applied methods for road safety and current applications and outcomes of urbanization in various parts of the earth's surface. Furthermore, additional approaches to road safety, risk management, and traffic accident and density analysis are added in the review article. Some importance of the road and LULC-related details analysis is to identify:

• Various uses of road safety analysis can support researchers and management teams in keeping an eye on the best practices for land use and change measurement, traffic congestion analysis, LULC forecasts, and road risk assessment.
• Vast areas of applications are noted in this review article, therefore researchers in the academic domain can identify the various approaches to the research activities and publications. Furthermore, they can compare every application in different aspects and choose the best application in a particular area.
• Urban issues in recent times are frequently increased therefore appropriate measurement of difficulties in urbanization is most needed. This review article can help them with appropriate approaches.
• Road safety measurement, traffic congestion, and network analysis like shortage distance, density analysis, point-to-point measurement, traffic volume measurement, and hotspot analysis can help the understanding of the various methods in a single review paper.
• Many approaches are added in the single review papers therefore, researchers can benefit from this review article with lots of applications.
• Various datasets-related applications like MODIS, Landsat, Sentinel, LISS-III, and DEM are noticed in this review article.

### 1.3 Research motivation and aim

Urban infrastructure mostly depends on the availability of road networks, urban amenities, and the economic stability of the area [12]. Population pressure and anthropogenic activities generally decrease environmental health conditions and increase socio-environmental vulnerability. Thus, effective management, creative adaption techniques, and planning may support the implementation goal, and the growth of the road network is boosting accessibility to transportation at a minimal cost. Furthermore, high road network development increases agricultural land losses, air pollution, CO_2_ emission, and green space dynamics due to the highway development and built-up expansion can trigger heat waves, thermal variation, and land subsidence [10,[58], [59], [60], [61]]. This research review indicates that the road network is related to different investigations and applications for the analysis of road network study and relates to the LULC variation. Google Scholar platform was used for related literature finding where some keywords are applied like road network, density analysis, and road infrastructural development on LULC, road accident analysis methods, road safety analysis, road, and LULC relation, and LULC prediction method from the year of 2010–2022. The foremost research aim is to (i) discover the related literature published with the keywords on road-related investigation like accident-prone area analysis, road network analysis, and road safety analysis, (ii) the relationship between road network and LULC, and related literature for LULC classification and simulation. This review investigation can help researchers, scientists, policymakers, planners, and other administrative authorities with novel methods and approaches for road network-related claims and environmental degradation assessment.

Comparable relations were described aimed at Portugal, France, and Spain. Those countries' explosions like the urbanization extents associated with the network of railway augmented for example railways prolonged equal to the years of 1920s and diminished subsequently. On behalf of an analysis number, the relative population strength and the change of railway, in addition to its frequency, fluctuates transversely in diverse epochs. A different study team shed light on how much each of the 44 Dutch metropolises developed and how crucial the railway's expansion was from 1840 to 1890 [62]. Researchers' memorandum considerable vicissitudes happening the urban growth decorations since period toward period and explosion the variation happening the railway lines number incoming the metropolises were the diffident nevertheless important descriptive mutable aimed at the convinced decade's numeral. Most investigations validate that the innovative roadway substructure, particularly the chief roadway substructure, simplifies the rearrangement of the populace from the middle toward a margin. Investigative the influence of the regional roads on the US Metropolitan Statistical Areas sub-urbanization between 1950 and year of 1990. Investigation indicates the intelligence that the developments happening in the road organization fascinate the populace laterally with the roads and underwrite the fundamental city's populace deterioration [63].

Some researchers measured clustering-based spatial-temporal proceedings happening in the roadways system analysis and planned the kernel density estimation (KDE) technique of the spatiotemporal system analysis toward distinguishing accidents of the traffic hot spots area [2]. Another research group applied the upgraded network KDE by way of the stricture toward recognizing the accident-prone incidence opinions based on the beginning through applying the technique of the zero-inflated undesirable regression of the binomial model and cumulative frequency (CF) [44]. Researchers projected the enhanced network quality of the KDE algorithm by enhancing the detachment among proceedings and the intersections kernel density function [35]. Formerly, the negative binomial regression algorithm of the zero-inflated was applied toward the appropriate increasing occurrence circulation of the density estimation of the nuclear outcomes, like significantly developed identification accident-prone accuracy points. Another part is, that Ethiopia country is one of the smallest urbanized countries, uniform in the sub-Saharan normal [64]. Founded in 1758, the Middleton Railway is located in Leeds, England, and is the oldest operational railway in the world (https://en.wikipedia.org/wiki/Middleton_Railway). Manchester, England is home to Liverpool Road Station, the oldest station in the world. For the proper examination of ecological and environmental concerns such as biodiversity, temperature variation, ecological variation, LULC change, issues connected to forest health, and global change, high-resolution satellite imagery might be helpful [65,66]. In Jiangsu Province, China, where the temperature rose by 15.96 °C between 2001 and 2015 and the area under contraction expanded by around 282.06 km^2^ (23.91 %), some researchers have examined the changes in land use and land cover. In the Guishui River Basin in Beijing, China, constriction areas have grown by 7444 ha [67], The Ili-Balkhash Basin (IBB) in Kazakhstan had a rise in urban areas of 514.29 % between 1992 and 2014, but the quality of living declined by 0.69 over the same period [68]. From around 11 % in 1985 to 44 % in 2015, the surface area of Nanjing's municipal and urbanized regions increased [69]. Similarly, another investigation indicates that Greater Guangzhou had high built-up lands from 2001 to 2006 [70]. At Xiongan New Area, China, where Sentinel-2 data was used for the LULC classification in 2016 and 2017, several researchers used the data for LULC classification. There, 51.59 % of the farmland is covered [71]. A study using Landsat data from 2013 to 2014, 2015, and 2015 examined thermal fluctuations in the Yangtze River Delta [72].

### 1.4 Article review procedure

In this review analysis of different aspects of road construction and related issues of LULC, applied approaches of road construction-related advantages and problems, LULC prediction models, datasets applied for LULC classification, and infrastructural development-related analysis were studied in detail, and a review of related literature. Utilizing the Google Scholar and Web of Science databases, this review inquiry employed the English language in evaluated articles from 2010 to 2020 for various dual elements, such as material linked to roads and LULCs. Different keywords are applied for searching the related important literature from those two databases, like transport infrastructure, road/rail, road safety analysis, traffic accident and spatiotemporal analysis, LULC change, LULC prediction, Satellite datasets, and infrastructural development. More than 370 papers were checked and analyzed for this review investigation. After that, some articles were removed from the literature study due to the non-English and duplicate articles, and 295 articles were selected for further analysis. After that, those articles were reviewed based on the abstract, keywords, and method applied for the analysis of road and LULC-related examination. After this stage, 152 articles were removed for similar methods and the same analysis, therefore 85 final articles were extracted for this investigation and 58 articles were further removed based on the research's significance, novelty, and areal distribution. The urban areas are gradually increasing and related problems are also rising with more disadvantages in the urban and peri-urban locations, therefore proper analysis of those issues like road safety, traffic congestions, and network analysis like shortage distance, nearest amenities, and nearest metro/rail/bus/car stations. Furthermore, LULC classification techniques and prediction models are also discussed with proper methodical and statistical definitions. Urban areas are more heterogeneous, therefore the road construction, residential areas, and alteration of LULCs need more need to be analyzed because of the fluctuation function of land area. Road congestion, traffic jams, LULC modification, and network analysis are all targets of anthropogenic triggers and population compression.

## 2 Literature review

### 2.1 Road-related application

**Fig. 2:**
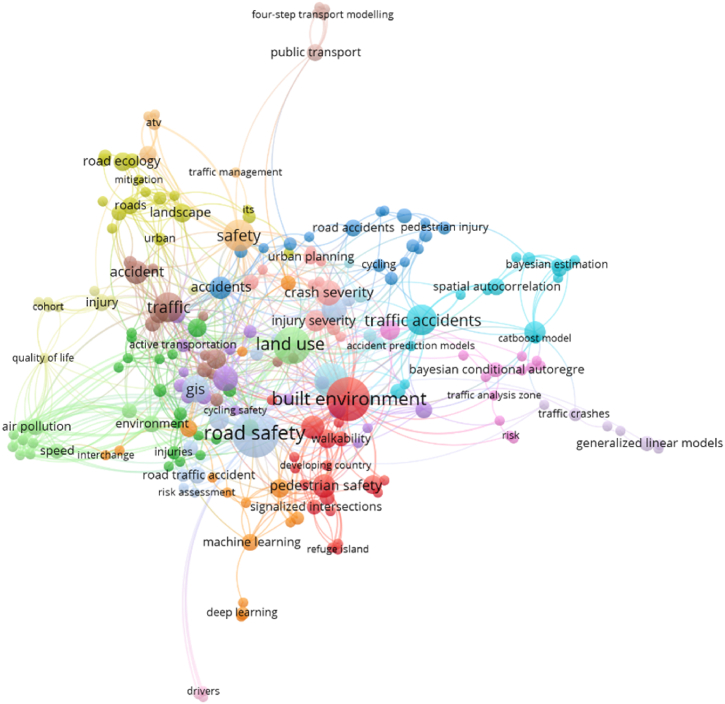
Road-related applications in different aspects (Based on the Scopus database).

Constructional works are gradually increasing because of urbanization and extraordinary populace concentration also triggering building contraction in urban and fringe areas where mostly landforms are rehabilitated into the settlement [66,73]. Different parts of the earth's crust are facing green space damage, reduction of water bodies, soil moisture loss, and temperature increase, and most of the parts are located in land subsidence due to high-rise building construction. As a result, in order to link cities as well as various portions of the highly linked areas, road extensions are being created. National highways, state highways, streets, sub-streets, and railway lines are also gradually increasing with the help of novel technologies and approaches [74,75]. Hilly regions of the altered fragments of the earth's crust also develop railway and road contraction. Founded in 1758, the Middleton Railway is located in Leeds, England, and is the oldest operational railway in the world. (https://en.wikipedia.org/wiki/Middleton_Railway). Liverpool Road Station is the world's oldest station situated in Manchester, England. The first known road contraction was found in Mesopotamia, which is currently in Iraq. Additionally, Joseph Mitchell pioneered the development of contemporary concrete roadways in the years 1865–1866 (https://www.fhwa.dot.gov/infrastructure/). Therefore many techniques are applied for road contraction in different parts of the world with low-cost contraction and variation of road lines [76,77] road contraction (Table 1). GIS methods are also used to obtain information on road contraction, such as information about bridge contraction, places that would be good for highway contraction, study of the length of the road, and measurements of the road's state [1,32,78]. This study includes road-related applications to determine global applicability. This review identified the Scopus database based on keywords like road safety, traffic accident, built environment, and GIS. Based on the published literature mostly examining keywords are used in this study (Fig. 2). In recent times, drones have also been applied for areal condition measurement, road health analysis, congested area identification, and traffic accident measurement. Several researchers have approved dissimilar spatial traffic accident investigations established on the GIS knowledge [79,80] traffic accidents. Initially, the greatest instinctive method for the measurement of traffic safety is through traffic accident frequency. Additionally, the majority of the previously available material is based on this assumption and focuses on identifying accident areas. The traffic administration section, conversely, pays additional consideration to the accidents that reason of thoughtful fatalities in the actual traffic organization [81]. As a result, it's important to look into how the provinces with the highest accident harshness are distributed spatially. Furthermore, the roadway system concentration influences accident concreteness and is not measured during density investigation [3,4,82].

**Table 1:** Indicates the road-related application and some literature review based on the previous peer-reviewed publications.

Reference	Application	Publication Country	Analysis
[2]	Traffic accident and road safety analysis	Spain	The accident recorded around 23.2 % of the roadway's sections (103 noticed while the entire roadways are 444)
[42]	Traffic accident-prone area analysis	Australia	37 % located all cyclist-related accidents. This might contain approximately 17 % of altogether fatal-related accidents, this result indicates higher than the average values of accidents.
[80]	Impact of traffic congestion on road accidents	United Kingdom	A robust procedure has been established toward allocating the accidents of M25 against its sections and the congestion index (CI) has been applied to characterize the traffic congestion level happening in every section.
[6]	Roadways traffic accident (RTA) applying the GIS knowledge	Turkey	The spatial analysis presented that the RTA-related death risk is higher in the rural parts with minor population densities than in more densely populated urban parts.
[83]	Spatiotemporal distribution of road accidents in Haryana	India	Death rate and injuries have enlarged by around 1.5 times throughout the previous two periods.
[32]	GIS-based traffic accident-related hotspots applying spatiotemporal statistical investigative	Vietnam	The RTA delivery hotspots have diverse completed periods bestowing toward dissimilar specific and season intervals of the daytime.
[84]	Road safety risk evaluation	Belgium	This should be understood the UK and Malta are double finest accomplishment nations subsequently those attain the optimum competence notch of one in a DEA-RS procedure, although around 25 nations are deliberated to remain underachieving
[85]	Road traffic accidents (RTAs)	Nigeria	In Nigeria, there is more overlap between the Northern and Southern States according to the RTA severity geographic study.
[86]	Vehicle influence in contradiction of permanent road equipment	Poland	The quickening sensor was situated at the gravity centre (protuberance around 700002). Gravity middle in the worldwide coordinate organization is: At Z, 478 mm, X, 1708 mm; and Y, 16 mm. The vehicle mass is 878 kg.
[87]	Road Safety Risk Assessment	High-Income Asian Countries	After the DEA analysis, the road accident risk datasets indicate that safety-related performances are needed for the different countries.
[1]	technique for distinguishing high-crash possibility roadways analysis with GIS	Iran	Using the investigating outputs system projected compactness during the course of the three investigation years 2006–2008, the suggested request approach was shown for the Arak-Khomein regions rural highways in the Markazi province, Iran nation, and hazardous steadiness segments.
[88]	Measuring road safety influences of teleworking strategy	Belgium	The outcomes demonstrate the amount for total areas like Car–Car Fatal–Unadorned Damage, and the Car–Car Unimportant Damage smashes are simulated to reduction through 173.4 and the 1199.8 components correspondingly completed a historical of the 4 years (2.84 % and the 2.84 %).
[89]	Distinguishing traffic accident clusters through the KDE network with the spatial statistics of the local area	USA	Exceptional spatial problematic situation comparable coincidences sideways the roadways system, through the roadways enormous amount sections (84,030 in the investigation), nonetheless actual insufficient (2637) foundation coincidence sections, the reproduction consequences strength might be considerably altered among this complete, and design haphazardness.
[90]	A KDE technique on behalf of the roadway systems	Japan	Street linkage contains 35,235 relations and around 25,146 nodes, and the entire distance is around 2190 km. Locations amount anywhere accidents of the traffic happened happening the street linkage is around 3109.
[91]	KDE on behalf of the accidents based on traffic in a linkage space	USA	Distinguishing the related boundaries customary 2-D planar of the KDE approaches happening the linkage space, the manuscript matures the network of KDE methodology toward describing spatial designs of the accidents of traffic on the roads.
[43]	Black Spots on the Highway with KDE	India	During the investigation, seven obscure spots were recognized in the Gurgaon-Jaipur Segment of the highway NH-8 (at km 186.980, 149.740, 125.240, 121.00, 71.580, 63.100, and 60.920) through the KDE technique.
[92]	Detect hotspots based on the GIS	Canada	This investigation presented a double-stage method for recognizing the hotspot positions inside the road linkage. The planned methodology combined the GIS-oriented linkage KDE examination through the HSM linkage transmission technique
[21]	The severity Index (SI) in the influences through roadways-related security barricades analysis	Australia	Simplified explanations like trial 4 were exposed to be statistically significant by the = 0.05 level: Ffit = 166.65 > Fcrit (2, 77, 0.05) = 3.12 (p < 0.001).
[93]	Relentlessness Prediction of the Traffic Accidents	Malaysia	An RNN procedure accomplished the finest authentication with 71.77 % accuracy once associated with a BLR and the MLP simulations.
[94]	Traffic safety assessment for the traffic analysis regions	Colorado	The total crashes number on behalf of the 46948 for 733 TAZs, amongst fatal crashes is 0.32 %.

Accidents in the roadway are singularities that can necessitate intense sympathetic of together the spatial and temporal mechanisms instantaneously [95]. Like, when dualistic or additional accidents happen at an adjacent spatial juxtaposition but fluctuate in their timing, they might not probably characterize an important cluster. Similarly, two different accidents that happened in some similar period historically but fluctuated spatially are correspondingly not probable to characterize an important cluster [96]. Henceforth, solid knowledge of the temporal and spatial magnitudes is essential for the successful appreciation of the hotspots, and eventually manipulative improved accident-prone areas mitigation approaches [38]. Conversely, comparatively few investigators have determined happening the spatiotemporal investigation aimed at analysing the clusters of the accident-prone. This technique can be applied to discover the accident datasets at methodical time intermissions like, the particular day hours, and existences of a particular year month, or week [78]. It works through principal sub-dividing the accident datasets according to the time of the manifestation established on the selected intermission, while is formerly monitored through the presentation of the examination of the spatial procedure like KDE [35,42,44].

### 2.2 LULC-related application

**Fig. 3:**
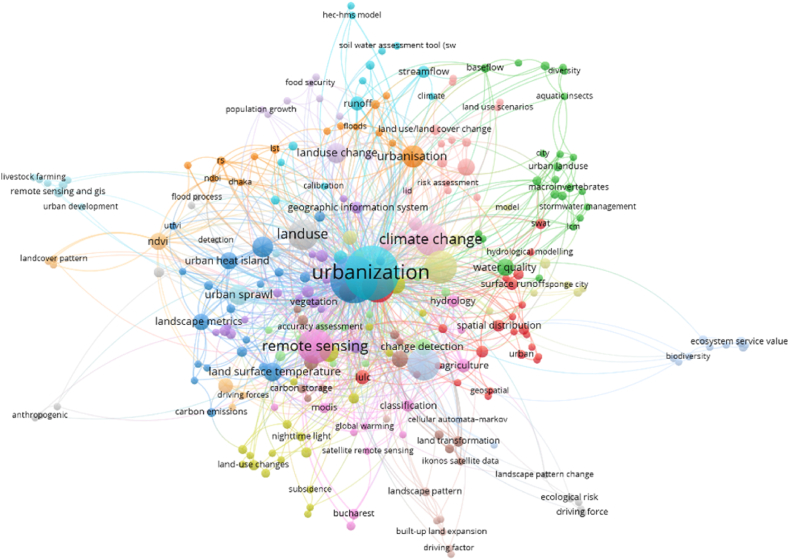
Land use-related application (Based on Scopus database).

**Fig. 4:**
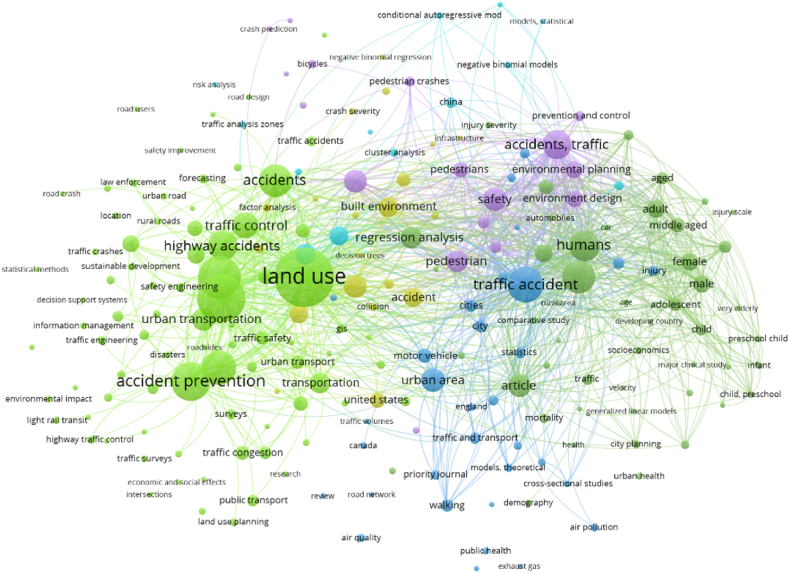
Road and urban land use-related works (based on Scopus Database).

Important junctures in the LULC's history and structure can often be characterized by notable events since human activity has left a lasting mark on the environment and backdrop that has been discernible for thousands of years [97]. Consequently, the present configuration and procedures of the land covers are a portion of the inheritance from the historical, and investigating historical LULC alteration is important to appreciate contemporary human-environment alteration and to forecast the expansion of the future LULC change [41,98]. LULC is noticeable in the remotely sensed datasets from the satellite platforms, though it necessitates understanding the ground-truthing datasets and uncertainties would continue [99]. LULC was altering paint happening like natural and human arrangements work together. Sympathetic numerous issues manipulating the LULC change have been the scientific concentration investigation transversely numerous locations, disciplines, and scales. Nevertheless, the shortest capacities alone are not enough to deliver a thoughtful of the services driving alteration. Connecting explanations at an assortment of temporal and spatial scales to experiential models delivers a complete method for understanding LULC change [100]. GIS-oriented methods toward the LULC appropriateness examination have origins in the hand-drawn submissions of overlap methods applied by the landscape architects of America in the nearly nineteenth and early 20th century [101]. LULC suitability investigation is additional than the GIS-based process also this approach encompasses participating methods [102]. While geographic information systems and GIS databases are important tools for organizing activities, organizers also consider influence associations, constituencies, complex municipal locations, and provincial challenges. LULC is one of the main concerning factors for landforms alteration and this study not only correlated with the land change but also related with the transportation analysis. Therefore, different applications of LULC are essential to identify the models or algorithms used in LULC analysis. The most widely used models and algorithms are found using keywords derived from Scopus. To find LULC-related analyses worldwide, use the keywords LULC, urbanization, remote sensing, and climate change (Fig. 3). These appeals on behalf of the socio-political standpoints apply a GIS as a tool aimed at forecasting. Subsequently the various initiation modeling methods in the RS and GIS technologies, investigations were completed by applying the approaches to investigate the LULC alterations and assistance to develop urban growth measurement strategy and approach [16]. Though the demonstrating methods have extensive variability in their theoretical beginning, the Geo-simulation methods have become an additional consideration currently relative to the GIS development and RS technologies [41]. Previous analysis correspondingly exasperated to discourse the difficulties applying numerous categories of the Geo-simulation techniques like cellular automata [103,104], Markov chain [105,106] Markov chain, and Multi-agent systems. The LULC classification, analysis, and prediction have many applications because changing climate and anthropogenic activities are gradually damaging the natural environment and the surrounding phenomena are also affected due to the LULC change [41]. Some applications are recorded in the previous studies like area change analysis, vegetation cover change [41], forest dynamics analysis [97], water area measurement, drought area analysis, green space measurement in urban areas [107], soil moisture analysis [108], thermal variation measurement [109], ecological diversity analysis, urbanization, or urban growth measurement [15], future urban growth analysis, future forest area analysis, flooded area measurement, future prediction of LULC using different simulation models, and planning the future scenarios (Table 2). Since there is a simultaneous correlation between roads and LULC at the urbanization point, it is more important to analyse applications connected to both land and roads. In this study road and land use-related analysis are used for identifying the worldwide applications. The Scopus database is searched using terms relating to roads and LULC, including traffic accidents, road safety, climate change, remote sensing, and GIS. Based on the data, land use, and road accident analysis are mostly examining in worldwide (Fig. 4).

**Table 2:** Indicates the LULC-related application in the literature review in the world aspect.

Reference	Application	Publication Country	Analysis
[98]	LULC changes study with the evolution rate simulation	India	Outcomes of the forecast approach discovered that around 14.7 % of the city's settlement parts might grow through the year 2019, 15.7 % through 2022, and 18.68 % through 2031.
[110]	LULC alteration by GIS-oriented procedures	Pakistan	Agricultural Area was also increased from 10,336 ha (11.49 %) in the year 1992 to 29,000 ha (32.23 %) in 2012 Settlement portion improved from 16,281 ha (18.09 %) in the year 1992 to 51,039 ha (56.73 %) in the year 2012.
[111]	LULC Classification and Topographic Correction in Yangjia River Watershed	China	Kappa value of individual 0.01 and Overall accuracy of individual 1.09 % advanced while that of arrangement 1, then suggests the established happening DEM assembled since 1:50,000 scale topographic diagram the SCS alteration might individually recover the arrangement outcome somewhat associated through that established on DEM (SRTM).
[70]	LULC analysis and weather variation impact assessment on the megacity's green spaces	China	Throughout a debauched metropolitan development and the modification historical, the NPP malicious assessment touched 331.5 g C·m−2 ·a−1 in 2001, 366.3 g C·m−2 ·a−1 in 2003, and transformed toward 273.2 g C·m−2 ·a−1 in the year of 2006.
[69]	Assessing a city scale LULC changes analysis using RS data	China	Settlement areas of Nanjing are observed at 11 % in the year of 1985, and 44 % in the year of 2015.
[112]	Previous LULC analysis with the help of CA Markov Chain algorithm	Allahabad, India	The city's population increased gradually by 18.12 % in the year of 1990, and 24.74 % in the year of 2011.
[113]	LULC prediction	Dhaka mega-city, Bangladesh.	The last 10 years have seen a 24 % rise in settlement, measured in hectares. In 1990, there were 11.696 ha, and in 2000 there were 14641 ha.
[98]	Statistical procedures based on LULC alteration and land features growth analysis	Gautam Budh Nagar area, Uttar Pradesh	The simulation indicates that the settlement areas increased by 15.7 %, and 18.68 % in the years 2022 and 2031.
[114]	Bannerghatta National Park's Forest Areas Dynamics estimation	Karnataka, India	Cities had parks with an approximate 5–6% increase in 1992, and between 1999 and 2007, there was an approximate 5–10 % increase in afforestation.
[115]	CA-Markov Chain Model, Markov Chain, Logistic Regression for LULC prediction	Lucknow, India	Settlement area located 53.60 km^2^ in the year of 1993 and 302 km^2^ in the year of 2013. The growth rate is 4.6 % and 26 % in the years 1993 and 2013 respectively.
[116]	Metropolitan LULC dynamics Prediction by applying the CA-Markov	Phuket Island, Indonesia.	The outcomes indicate that the urban growth from 2014 to 2026 was increasing around 4010.31 ha.
[117]	ANN based cellular automaton simulation for LULC	North Sumatra	LULC simulation observed for the year 2050 located 4 % of plantation land increase while cropland and forest areas will increase by 1.6 % and 1.2 % by 2050.
[103]	Megacities LULC alteration and simulation of the expansion	Nagpur City, Maharashtra	Settlement is located at 41.24 % and 67.86 % in types of 2000–2020, while the green space decreased by around 15.93 % during 20 years.
[118]	Predict the city's LULC dynamics on logistic regression and CA	Hyrcanian region, Gilan, Iran	The settlement was increased from 36012.05 ha to 59754.8 ha from 1989 to 2013 like 1.7 % of the total land.
[52]	CA and AHP-based city expansion measurement	Qazvin City, Iran.	This approach practices an appropriate technique on behalf of penetrating and choosing landforms aimed at development. The realized approaches were applying data on behalf of the year 2016 to simulate the expansion of the year 2026.
[10]	Urban expansion analysis with the help of heat variation and urban heat island (UHI)	Kolkata, India	Subsequently, human happenings were improved in this area and correspondingly, the built-up area has increased. Everywhere 24.324 % of the part has been intensified.
[119]	Object-oriented crop classification	Northwest Sheridan County.	An approach of mid-season had a κ statistic of 0.63 and overall accuracy is around 78.4 %.
[120]	Simulate the flood-prone zones with the help of numerous approaches	Kendrapara, India.	The impact of the SPI on the incidence of floods was demonstrated by the frequency ratio (FR) analysis. The percentage was at its maximum (1562), and the SPI was between 0 and 230,328, with a monitoring range of 690,985 to 1,151,642.
[106]	Hybrid-based GEOMOD-Markov Chain algorithm of LULC dynamics	Sufichay River catchment, Iran.	At a 200-m spatial resolution, the percentage of the study area that consisted of Misses, Hits, and False Alarms of simulated orchard gain during the period of 2000–2015 was 1.84 %, 0.45 %, and 0.74 %, respectively. Based on the limited extent of simulated orchard gain and actual orchard cover across the study region (1.2 % and 5.3 %), the FOM for these events was noteworthy at 15 %.
[121]	Spatial modeling and justification of the Forest green dynamics	Kanakapura Region, India	The authenticated diagram was originated to be observed as 84.26 % precise. The authentication is also verified applying the ROC methodology which was originated to be around 0.614. The algorithm was then additionally protracted to simulate forest losses for the year 2015.
[122]	LULC dynamics established on the landform variation modeler	Muzaffarpur city, India.	A Multi-Layer Perceptron (MLP) neural net as designated by Rumelhart et al., 1986 is unique and is usually applied to ANN. The MLP-ANN training is founded on the Backpropagation (BP) procedure which is an administered exercise algorithm.
[123]	A land Change Modeler applied to simulate the LULC	Ganga River Basin, India	30 % of the settlement areas are increasing throughout the study periods.

### 2.3 Relation between land alteration with road development with GIS

Assumed the high death risk and damages caused by roadway accidents, the GIS methods were performed for example the important tool behalf of recognizing the roads accompanying the most accident hazards with added visual authorization of the accident constellations happening in the diagrams [42,124]. These investigative examinations concentrate on recognizing and examining the positions and the times that position the high accident risk for elderly inhabitants applying GIS-oriented spatial, temporal, and spatiotemporal approaches. The primary phasing of these changes is considered noteworthy due to the extensive participation of review articles. After the current literature preview with the review, the resulting approaches are applied: (a) Spatial examination through compactness ratio and KDE examination, (b) Temporal examination applying spider diagrams, and (c) Spatiotemporal examination through a comp/severity index/hotspot analysis technique [32,74,86]. Road traffic accident displays are intended to process clusters occurring in geographical time and space, and they do so by separating the temporal and spatial embellishments. A wide range of knowledge and investigation of these designs is consequently dangerous for the unindustrialized suitable accident deterrence approaches. Through the worldwide climatic changes and unnecessary urban development in certain parts, management of the risk and construction sturdier flexibility competence like the megacities essential profound important vulnerabilities sympathetic in a megacities culture and cautiously deliberations toward the Indigenous megacities physiognomies [125]. Novel GIS technologies [92] and communal GIS intelligence in the multitude obtaining form evidence volunteered, and harvest geographic information distribution would be combined interested in the decision-making of an entire risk management development [126]. It has been extensively acknowledged that investigation in GIS has been an amalgamating strength. Fundamentally, GIS is an interdisciplinary part and negotiation philosophy on or after cartography, sociology, geography, and computer science. Conversely, in preformation interpretation, GIS presentations in the numerous characteristics of the cities are disjointed and have altered topographies [45].

### 2.4 Section analysis

This review section has two different aspects of the analysis of the road and urban LULC change-related investigation and examining the suitable review process for the road-urban problems and issues, which are triggering factors for environmental degradation, life losses, high traffic accidents, traffic congestion, and other issues like future land dynamics, urbanization effects, urban amenities and planned building construction on the particular land. The first section shows the variety of uses related to roads, while the second discusses the global application of LULC in various regions of the world. We conducted these literature-cum-review articles to understand the different applications, approaches, analyses, and outcomes investigation in the same domain. Road contractions are also expansion to associate one city to another city, and different parts of the highly connected locations. National highways, state highways, streets, sub-streets, and railway lines are also gradually increasing with the help of novel technologies and approaches [74,75]. Consequently, numerous procedures are applied for road contraction in worldwide different parts with low-cost contraction and road lines variation road contraction [76,77]. GIS procedures are similarly applied for road contraction-related evidence such as suitable sites for highway contraction, bridge contraction, road conditions measure road length analysis, and using GIS techniques [1,32,78]. The aforementioned examination consistently exasperated to discourse the difficulties using frequent groupings of the Geo-simulation techniques like the Markov chain [105,106], cellular automata [103,104], Markov chain, and Multi-agent system. Other LULC change study techniques that have been used by certain researchers to examine land modification include Random Forest (RF), object-based classification, supervised and unsupervised classification, and many more.

There are certain limits to this part since we are unable to analyse the entirety of the publication in this review article due to the large number of research and review papers that are released every day. Some selected articles were chosen for this section while the same research method applied articles were made in the same section. The majority of the papers were on road-related urban issues, and we carefully choose and analyse the many and diverse ways that have already been used for various regions of the earth's crust. Road dataset information is limited therefore lake of road datasets can increase some problems like road construction variation measurement, and traffic accident areas need more attention to reduce those kinds of phenomena. More attention needs to be paid to the various sections that specifically analyse the diversification of the road-urban application using various methodologies. Each section, such as the ones on network measurement, LULC classification, road accident analysis, and LULC prediction, has some limitations because the data source is not readily available. Future studies might look at how road modification and LULC prediction vary, potential LULC classification techniques where heterogeneity can be enhanced and developed using cutting-edge machine learning algorithms, and statistical data analysis models.

## 3 GIS application on the road

### 3.1 Road network analysis

#### 3.1.1 Density estimation

Analysing the density of road areas or transportation in certain locations or cities is increasingly crucial when it comes to road networks. The study examined the use of both point and line density analysis in determining the road network density and traffic accident point density, respectively [127]. To divide certain cities into many small right-angled cells using the neighbouring measurement of d (which conforms to a pixel-oriented component continually a completed GIS maps), for instance, the examination of the point density of accident as the examination of principle goes. The density of provincial accidents characterized by the cell *k* is *Drk, Dak* is denoted that the density of road network, neighbourhood radius is set towards *ρ*, *Nk* (*ρ*) represents the number of accidents happening environs through a centre of the cell *k* by way of a point and *ρ de*noted by way of the radius. *Lk* (*ρ*) represents the road measurement in an equivalent neighbourhood, where *Drk* and *Dak* are investigated by Equation (1).

(1) $$\left[D_{1}^{a}{\;D}_{2}^{a}\ldots ..D_{k}^{a}\right]=\frac{1}{{\pi \rho }^{2}}\left[N_{1}\left(\rho \right)\;N_{2}\left(\rho \right)\ldots ..N_{k}\left(\rho \right)\right]\left[D_{1}^{r}{\;D}_{2}^{r}\ldots ..D_{k}^{r}\right]=\frac{1}{{\pi \rho }^{2}}\left[L_{1}\left(\rho \right)\;L_{2}\left(\rho \right)\ldots ..L_{k}\left(\rho \right)\right]$$

Finally, the researchers obtained an accident-prone dispersion map of density from the density calculation, which targeted each cell using Equation (1). The investigation goals for road linkage concentration must be replaced separately after the locations along the road segment that are prone to accidents. Most of the above listed inquiries on road accidents focused on the frequency of accident sites [128,129]. According to the reported research, each accident point weight is assigned a severity rating. Prior to determining the severity of the traffic accident, the road density must first be examined in order to determine how harshly the road density distributes the severity of the accident. Occupancy of the neighbourhood where the most serious disaster occurred of the cell *k* be *xl*, *l* = 1, 2, …, *Nk* (*ρ*), where the accident importance severity density (SD) are denoted as *Ds k* of cell *k* is Equation (2).

(2) $$\left[D_{1}^{a}{\;D}_{2}^{a}\ldots ..D_{k}^{a}\right]=\frac{1}{{\pi \rho }^{2}}\left[\sum_{l=1}^{N_{1\;\left(\rho \right)}}x_{l\;}\sum_{l=1}^{N_{2\;\left(\rho \right)}}x_{l}\ldots ..\sum_{l=1}^{N_{k\;\left(\rho \right)}}x_{l}\right]$$

#### 3.1.2 Cluster analysis

The road-based Cluster examination mentions an examination procedure that boundaries an established of abstract or physical substances into dissimilar groupings composed of comparable substances to complete convinced instructions. Clustering investigation of the spatial features is constructed on the organization of the instructions based on the relationship of the convinced three-dimensional features, consequently for example to acquire characteristics of the three-dimensional circulation it must have related objects [130]. A non-aggregate connotation of the investigations is that overall calculations are constructed on the datasets with sample point attributes of a solitary accident, never the complete datasets with characteristics subsequently the spatial accumulation [131]. This is supplementary all-inclusive to pronounce an accident point spatial distribution characteristics [132]. Outlier investigation controls the relationship between a neighbouring opinion and the option point in an interplanetary through investigating a local Moran index *I* (Local Moran's *I*) data point statistic [131]. The Moran Index is calculated based on equation (3).

(3) $${\;I}_{i}=\frac{x_{i}-\bar{X}}{S_{i}^{2}}\;\sum_{j=1,j\neq i}^{n}w_{i.j}(x_{j}-\bar{X}$$

where ${\;I}_{i}$ is indicates a local Moran's I datasets point statistics, $x_{i}$ and $x_{j}$ are representing that the dataset attributes point *i j*, *n* is denoted by the total number of data points, and X is denoted by the worldwide attribute average, $w_{i.j}$ was recognized that a three-dimensional weightiness among datasets opinion *i* and other datasets points *j*, frequently occupied by way of the opposite of the detachment among the dual altered opinions variables, and the *s* indicates that a second-order example overall point moment attributes of datasets excluding the datasets points. equation (4) is applied for the *S2 i*:

(4) $$S_{i}^{2}=\frac{\sum_{j=1,j=i}^{n}\;{\left(x_{j}-\bar{x}\right)}^{2}}{n-1}$$

While *z* score ZIi of datasets point *i* might be estimated by Equations. (5), (6), (7)).

(5) $$Z_{Ii}=\frac{I_{i}-E\left[I_{i}\right]}{\sqrt{V\left[I_{i}\right]}}$$

(6) $$E\left[I_{i}\right]=-\left(\sum_{j=1,j\neq i}^{n}\omega_{i,j}\right)/\left(n-1\right)$$

(7) $$V\left[I_{i}\right]=E\left[I_{i}^{2}\right]-E^{2}\left(I_{i}\right)$$

The commonly applied arithmetical consequence confidence is around 95 %, where p values are around <0.05, this might be measured as statistically substantial. A conforming threshold value like the *z* is denoted by the ±1.96, in the normal distribution. Under statistically significant circumstances, the uncertainty of the values of the *I* is progressive, it designates that a dataset point has the equivalent least or most characteristic values, for example, a neighbouring point, and that points are the parts where a low–low-value cluster or a high–high values cluster. Where the values of *I* are undesirable, this indicates that there is a significant alteration among the adjacent point and the attribute values of a dataset point, which was a point of the outlier.

#### 3.1.3 Point-to-point analysis

**Fig. 5:**
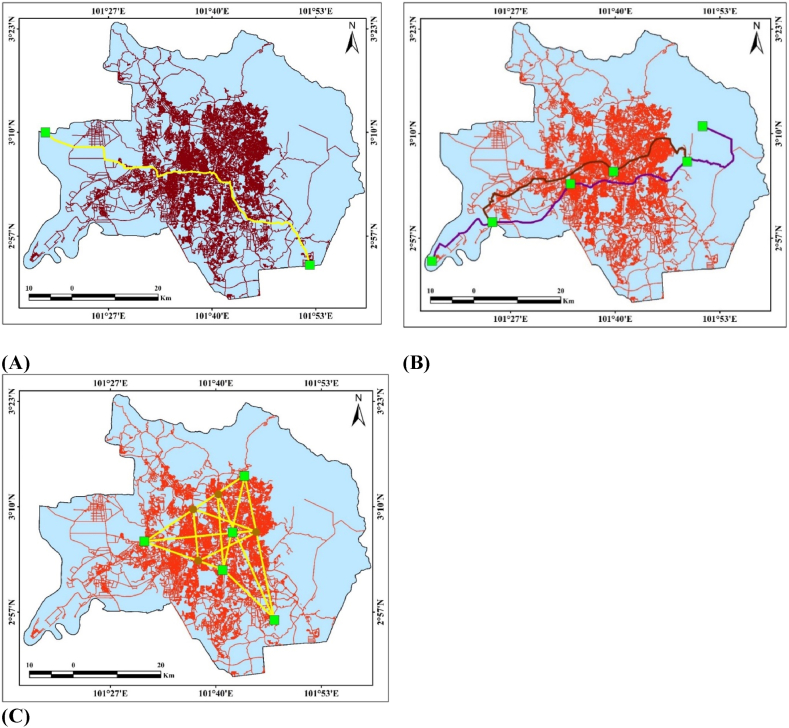
(A) Point-to-point observed roadways network analysis in GIS, (B) Optimized fleet analysis technique, (C) Origin-Destination-OD cost matrix analysis of the essential aspect. The Grater Kuala Lumpur, Malaysia is used for those analysis.

The most crucial technique for road network research and other road-related tasks is point-to-point analysis. Point-to-point analysis enables several crucial techniques, including the quickest route identification, shortfall distance calculation, and nearest-neighbor determination (Fig. 5a). Due to the region's high level of development and abundance of large-scale road building, the study generated applications connected to roads using Malaysia (Kuala Lumpur and adjacent areas) and Spain (Madrid). To identify better solutions for urban development like shortage path analysis, road accident analysis, hotspot, and point-to-point analysis.

A. **Find nearest** is the technique where searchable places like ATMs, banks, malls, bus stands, railway stations, and many more places are located using the network analysis tool.
B. **Shortage Distance** is an alternative approach in which the network analysis techniques are used to estimate the shortest path or road from one location to another. The shortest distance analysis and several routes to the destination are often determined using the shortfall distance.
C. **The fastest route** is another method where those roads are selected which take the lowest time to reach one place to another place. This technique is included in the account of vehicle speed limits, road conditions, road classification, and other costs to determine the least travel time.

A Point-to-point observed roadways network examination is also included in different scales like cycling roads; eco-friendly scenic, emergency vehicle routes, and other related applications.

#### 3.1.4 Finding coverage

Finding coverage is one of the essential network coverage analysis applications where service area-related results and other activities are identified, like fire station coverage, ATM, Hospital, and bank coverage areas identification. This technique was applied like one fire station coverage areas are identified 5, 10, 15, 20, and 25 min from the station, which defines the time taken for any emergencies or demand of the people.

#### 3.1.5 Optimize fleet

The optimized fleet technique is widely used in service-related activities like online shopping, food delivery, repair work, and any type of indoor service where salespersons can find the set of orders. This technique will help to reduce the overall operating cost due to low time purpose and manage the sets of vehicles and drivers. This technique is mostly used for finding the efficient route for repair, delivery, or any type of service (Fig. 5b).

#### 3.1.6 Select optimal site

Finding the right location for a fire station, hospital, retail center, bank, ATM, and market requires careful consideration of a number of factors, all of which are looked at with the use of a site selection tool. Additionally, several scholars have looked at site selection via the GIS method [[133], [134], [135], [136]]. The businessman may also implement the ideal shop location where the majority of the target market is present, such as in the middle of a residential area or next to a hospital, with the aid of the network analysis of the best site selection.

#### 3.1.7 Cost matrix estimation of origin-destination-OD

A GIS-based technology called Origin-Destination-OD cost matrix estimation determines and locates several places that are chosen according to people's needs and wants. This method makes extensive use of ArcGIS software (Fig. 5c).

#### 3.1.8 Vehicle routing problem

One of the most important prerequisites for looking into vehicle management is vehicle route analysis. One such statement addresses the most effective method of assigning specific consumer groups to a fleet of cars and setting up a schedule for their visits. The restraints are to comprehend the vehicle routes through the obtainable resources and inside the time restrictions compulsory by the driving speeds, driver work shifts, and customer promises. The Network Analyst delivers the VRP solver that can be applied to control the explanations for such multifaceted fleet administration responsibilities.

#### 3.1.9 Location-allocation

Location allocation assists you in indicating which conveniences since the set of facilities to function is founded based on their possible communication through the ultimatum opinions. It can help you to respond to those questions similar to the following:

A. The fire station, which fire station is nearest to the affected residential areas or locations, best response to the time-to-time.
B. Shopping mall, which shopping mall is near the density area or the demand location because density areas and demand location identification are necessary for fulfilling the customer importance.
C. Market or the building locations investigation to minimize the low distances, and high distributions, manage all the applicable opportunities and other aspects.

#### 3.1.10 Time-dependent analysis

Most of the explainers are recorded overhead permitting you to integrate the historical traffic data and live into an examination, consequently, you can discover the finest route for the assumed time/pay; discover the best habitation to preposition an ambulance for emergency purposes at 5:00 a.m., 10:30 a.m., 2:30 p.m., and so on. Also, this technique produces some service zones for different times/days (Table 3).

**Table 3:** Different types of road network analysis methods and published literature.

Approaches	References
Density estimation	[32,89,90,[127], [128], [129],^137^]
Cluster analysis	[42,84,89,[130], [131], [132],^138^]
Point-to-point analysis	[32,90,139,140]
Finding coverage	[[141], [142], [143], [144]]
Select optimal site	[32,135,136]
Origin-Destination-OD cost matrix	[145,146]
Vehicle routing problem	[32,86]
Location-allocation	[32]
Time-dependent analysis	[[147], [148], [149], [150]]

### 3.2 Road accident-prone area estimation

The most significant influence on behalf of road networks and connected studies is to identify the Road Traffic Accident (RTA) examination in the world. In the world road accidents were recorded around 50 million injuries and 1.3 million deaths due to the RTA and related activities (Table 4). Understanding the causes and locations of accidents is crucial for reducing traffic accidents and associated incidents. The high accident-prone area identification is more important to investigate the actual condition [33]. It's too difficult to identify the most accident-prone areas where accidents are occurring in selected periods [92]. Hotspot area identification is one of the important factors in estimating the current condition of the dense road traffic conditions area. The hotspot areas identification for road accident-prone areas has some limitations because of construction among time and space, lack of visualization, and priority of the hotspot ranking, with traffic volume not considered for this method [151]. A large area of accident-prone zone identification is more important to estimating the RTA database. The Hierarchical procedures comprise Clustering Using Representatives (CURE), Clustering by Hierarchies (BIRCH), and Balanced Iterative Reducing, which are operatively aimed at visualizing and summarizing the data. However, those techniques are problematic in scale-up subsequently every pronouncement requirement to estimate numerous events. Clustering In Quest (CLIQUE) and Grid-based algorithms include Statistical Information Grid (STING). The hierarchical and partitioning procedures are appropriate to recognize the spherical shape of the clusters. Ordering Points to Identify the Clustering Structure (OPTICS), Density-based Clustering (DENCLUE), Density-based algorithms comprise Density-Based Spatial Clustering of Applications with Noise (DBSCAN), and those are current procedures for recognizing clusters of arbitrary shape. A KDE technique might professionally diminish a consequence of the noise through correspondingly apportioning noise interested in the contribution datasets [42,43,89].

**Table 4:** Different types of road accident-prone area analysis methods and literature.

Methods	Reference
Kernel Density Estimation (KDE)	[1,2,32,42,43,[89], [90], [91]]
Maran's I statistic	[1,2,6,43,88,89]
NetKDE	[1,89,91,92]
Severity Index	[21,32,83,85,86]
Hotspot	[2,6,42,43,89,90,92]
Comap Method	[32,92]

The GIS-based traffic safety and accident-prone area identification are more important and those are already established for examination [1,33]. The accident-prone areas were deposited in a GIS-oriented dataset, where the investigators could easily recognize the explanations for every accident. GIS can be used for safety analysis where analysts can assemble, deposit, manipulate, interrogate, investigate, and visualize the GIS-based spatial data analysis. To explore the hotspot area, GIS-based spatial analysis tools are widely used in RTA [42,152]. The KDE technique is widely used for analysing the highly influencing accident-prone areas on road network analysis [92,152]. Some post-studies have used Some methods for studying traffic analysis and road accident-prone areas [42,74,153].

#### 3.2.1 Comap method

The comap technique is extensively applied for spatiotemporal analysis of road accidents [154]. This method was previously applied for the high rate of accident intensity analysis of specific periods [92]. The comap technique empowers us to investigate the spatial-temporal incorporation and assistances to comprehend the associations among the positions or places of RTA and their changes over time [154].

#### 3.2.2 Severity index

**Fig. 6:**
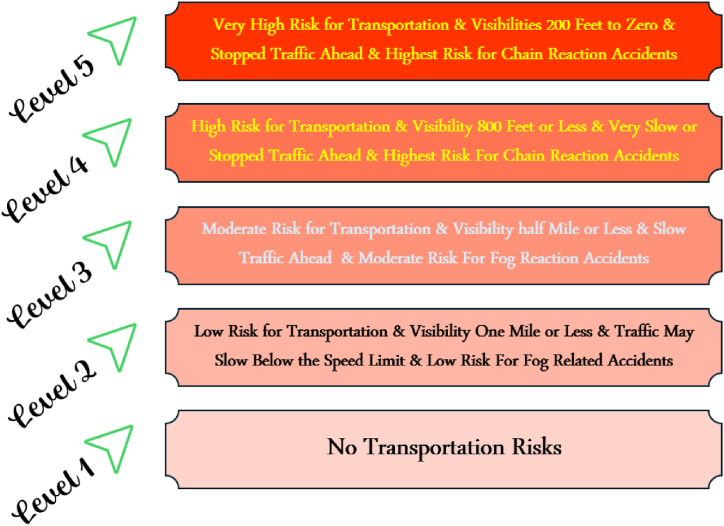
Scale of severity index (Source: https://www.weather.gov/hnx/HNXFogSI.html).

An approach to determining the significance of an event is the accident-prone Severity Index (SI). Determining whether the high/low cluster is occurring in the absence of biased datasets is undoubtedly hard (Fig. 6). The productivities of the accident severity or harshness investigation technique are diverse weighting classifications. This methodology provides advanced weights to examining the additional thoughtful accidents, nevertheless not through the tremendously high standards calculated unintended percentage to the charge of the accidents [21,74,85]. The investigation used an accident severity increment structure applied by the Belgian government. The research results indicate separate weights like 5, 3, and 1 were aimed at fatal serious, and slight accidents, correspondingly [155]. equation (8) is used for severity index analysis.

(8) SI=L+3S+5D

where the SI indicates the SI on behalf of every position; L indicates the entire number of slight injuries numbers; S indicates the entire number of serious damages; and D denotes the entire number of deaths.

#### 3.2.3 KDE analysis

Density data investigations were the main usage of the KDE. KDE is frequently used in spatial modeling for the investigation of accident-prone zones on roads. A smoothed surface of any category of the density estimation was produced by applying the KDE technique to a bandwidth investigation. The kernel purpose is exploited to apportion a weightiness to the section neighbouring the proceedings comparative towards the detachment toward the point happening. Following that, the point-event center was where the importance peaked, and the inquiry circle's range saw a smooth decrease to zero. Finally, by adding distinct kernels to an inquiry segment, a smoothly continuous concentration surface is created [42]. equation (9) is used for KDE in any area.

(9) $$f\left(s\right)=\frac{1}{{nh}^{2}}\sum_{i=1}^{n}K\left(\frac{d_{i}}{h}\right)$$

where f(s) is de f(s) is denoted that the density estimation of the particular section of s; n indicates an observation amount; h indicates an investigational bandwidth; while the K was denoted as a kernel function; $d_{i}$ is defined as the remoteness among the actual positions with an ith surveillance.

#### 3.2.4 Categorization of the hotspots

**Fig. 7:**
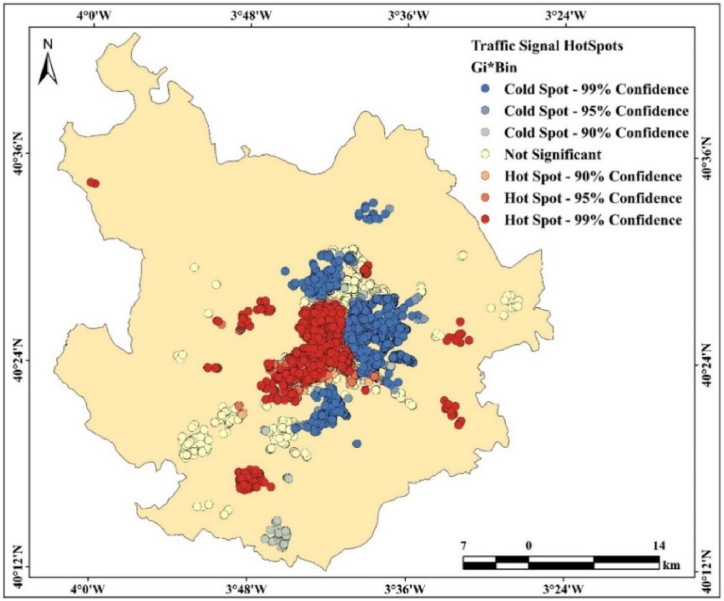
Hotspot analysis using point datasets in ArcGIS. The Grater Kuala Lumpur, Malaysia is used for those analysis.

Road accident or safety identification does not look at the hotspot locations, hence hotspot analysis is mostly utilized for highly impacted area investigations where real datasets are more essential. When very low, low, medium, high, and very high accident-prone zones are constructed, the hotspots create equal spacing between them [23,92,156,157]. Several software programs apply the polygon, line, and point datasets for hotspot analysis, and the hotspot region is manually determined (Fig. 7). equation (10) is used for hotspot analysis.

(10) $$G_{i}^{*}\left(d\right)=\frac{\sum_{j=1}^{n}W_{ij}\left(d\right)x_{j}-w_{i}^{*}\bar{x}}{s{\left[\frac{W_{i}^{*}(n-W_{i}^{*}}{n-1}\right]}^{\frac{1}{2}}}$$

where x is meant by the probability of spatial data; $W_{ij}$ specifies the spatial weightage of designated datasets which varies between j to i; $W_{ij}$ was identified the sum weightage like $W_{i}$; s is representing the x values standard deviation while $\bar{x}$ indicates a mean value of the selected datasets.

#### 3.2.5 NetKDE

The network KDE is the modification method of the KDE where 2D datasets and values are estimated [91]. The following equation (11) is used to calculate this method.

(11) $$\lambda \left(s\right)=\sum_{i=1}^{n}\frac{1}{r}k\left(\frac{d_{is}}{r}\right)$$

where λ(s) is indicates a density s; r is representing the search radius or bandwidth of a KDE; $k\left(\frac{d_{is}}{r}\right)$ is a weight of a point value i at a distance $d_{is}$ toward location s; also, k is denoted by the kernel function. Different numbers of the kernel functions are accessible, like Quartic; negative exponential, Gaussian, epanichnekov, and Conic. Established on the post-research identification, the kernel purpose excellent k indicates low significance rather than the excellent of the bandwidth search r in planar the KDE [89,158]. Some researchers [91] established a comparable condition in the NetKDE based on equations (12), (13)).

(12) $$k\left(\frac{d_{is}}{r}\right)=\frac{3}{\pi }\left(1-\frac{d_{is}^{2}}{r^{2}}\right)when\;0<d_{is}\leq r$$

(13) $$k\left(\frac{d_{is}}{r}\right)=0\;when\;d_{is}>r$$

In applying the NetKDE [91], recommended exhausting the linear section (named pixel) of the roadway transport as the elementary component on behalf of the manipulative density, accumulating coincidences, and visualization. Shorter portions are also more effective in displaying the distinctions across local subdivisions, according to research. A linear three-dimensional dataset is produced by a NetKDE by applying density standards to each pixel or linear segment. Other researchers also pronounced supplementary actions of the NetKDE application in their most current research work [159].

#### 3.2.6 Maran's I statistic

The other road accident-prone area estimation method is Moran's I statistic where the geographic objects are selected as dispersed, clustered, and randomly distributed [160]. This method is usually used to classify the geographical distribution and outlines and estimate areas that are prone to traffic accidents. The local Moran's I statistic was calculated from the Getis-Ord Gi∗ method while the covariance before the calculations was calculated. The following equations (14), (15)) are applied for Maran's I method estimation.

(14) $$I_{i}=z_{i}\sum_{j=1}^{n}w_{ij}\times z_{j}$$

(15) $$z_{i}=\frac{\left(X_{i}-\bar{X}\right)}{\sigma }$$

where n indicates the numbers of the spatial component or datasets of road accidents; $w_{ij}$ is denoted that the weighted values of a three-dimensional unit concerning i and j; $\bar{X}$ indicates a mean value of σ is representing a standard deviation (SD). A z-score and a p-value are calculated using this method.

### 3.3 Road safety and risk analysis

#### 3.3.1 Method for road safety analysis

Safety on the road is the most important factor for the investigation of the accident numbers, implementation, and adaptation strategies where road accidents must be reduced. The ‘Risk’, which is related to the two words like casualties and crashes, road safety is more important. For the road safety analysis, equation (16) is applied.

(16) $$Risk=\left(\frac{Road\;Safety\;Outcome}{Exposure}\right)$$

When the road segment performances were being produced, such as the number of trips, volume, and hours of vehicle travel, etc., the exposure was measured by various sorts of components. The country-level identification of the exposures is population density, the total number of vehicles registered, and km travelled by the passenger. Road safety performances are mostly affected by population pressure, road conditions, and vehicle speed limits, where awareness is also a triggering factor for road safety estimation [21,161].

#### 3.3.2 Roadways safety examination with DEA

The Road routine safety examination of roads or highways is a significant assignment for the safety of travellers. Safety performance analyse of the convinced characteristics, a standard for the instruments has continued as an elementary technique to be implemented by the investigators [[162], [163], [164]]. With the orientation to the functional procedures for this determination of the road safety analysis, the DEA will general procedure through its hypothetical source happening the linear software design. Researchers employed a linear sequencer to estimate an experimental construction equipment boundary (bench marking) on behalf of the primary era, fostering a better understanding of the DEA since the investigation's inception in 1978. A straightforward DEA algorithm, where the biggest performers are described based on the assumption that contributions must be minimized and productions must be maximized (e.g., in the field of economics). For calculating DEA, equation (17) is applied.

(17) $$\min\;R_{0}=\sum_{r=1}^{s}u_{r}y_{r0}\sum_{i=1}^{m}v_{r}x_{i0}=1\sum_{i=1}^{m}v_{i}x_{ij}-\sum_{r=1}^{s}u_{r}y_{rj}\leq 0,j=1,\ldots ..,nu_{r},v_{i}\geq 0,r=1,\ldots ..,s,i=1,\ldots .,m$$

where $y_{rj}$ and $x_{ij}$ indicates a $r^{th}$ production and the $i^{th}$ contribution correspondingly to a $j^{th}$ DMU, $u_{r}$ indicates the weight assumed through a t production r, and $v_{i}$ is a weight assumed to the contribution i.

Interpretation of the algorithm-oriented presentations on behalf of protection investigation in roadways and road protection situations were differentiated into 21 European countries [162], where perfect disturbance administration record scores were also calculated through the DEA. Additionally, using passenger travelled km, population, traveller vehicles for example contributions, and the fatalities figure as production, the DEA is applied on behalf of a risk level assessment of some republics [22]. Annual development monitoring for road safety is also supported by the application of the DEA approach [165].

#### 3.3.3 Roadways safety examination with ANNs

**Fig. 8:**
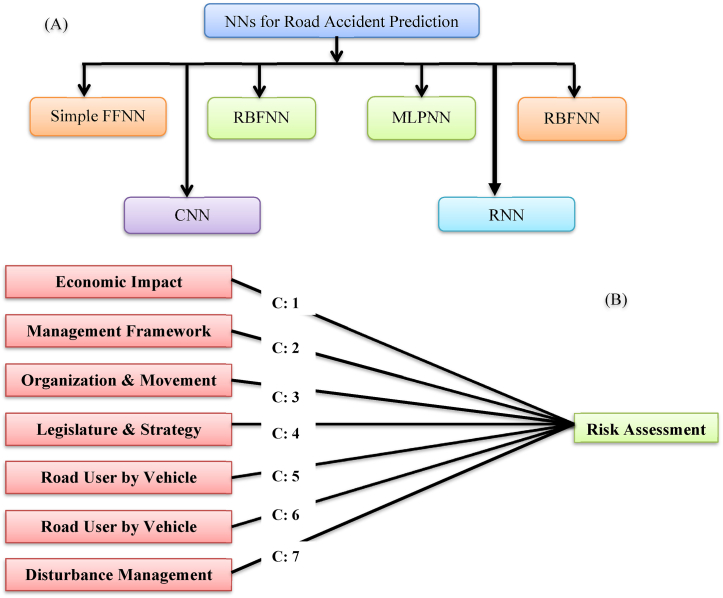
(A) Adopted NNs techniques for Road Safety and Accident Analysis [4], (B) Framework for road safety analysis [87].

The non-linear numerical statistics that were utilized to describe a complex correlation between the input and the output to pursue the embellishments were implemented using ANNs, a framework method. The ANNs have remained frequently instigated in numerous science fields for prediction purposes [37]. In the safety research on the road, the ANNs were used to examine the crashes through the reference to the roadway, vehicle, driver, and condition attributes [166]. The impact factors, such as a bright complaint, the driver's alcohol intake, and the driver's planned seatbelt use protection or safety, were evaluated following the application of ANNs. The ANNs were correspondingly used to control the correlation among model parameters and the crash severity by counting highway sections, years, and the degree of horizontal curvature, AADT, section length (km), heavy vehicles (percentage), perpendicular curving degree, with the summer season (proportion) (Fig. 8a).

Those results presented that the vertical curvature degree has a robust influence proceeding the number of crashes [167]. A previous study suggests that researchers can reiterate that ANNs were previously utilized, for instance, in an analysis model of the collision data, which was a useful process for examining topographies and geometry directed toward highways with additional causal implications on the safety of roadways (Fig. 8b).

#### 3.3.4 Algorithm with DEA and ANNs

A DEA amalgamation with the ANNs has not remained used happening the security of roadways, nonetheless, it's common now the additional grounds comparable corporate with bank subdivisions. Subsequently, an aforementioned research investigation it was determined that the DEA is commanding for the competence measurement, nonetheless on behalf of the simulation determinations the ANNs are forward, so a conversation happening afterward [168] on coalescing these two procedures to acquire the greatest conceivable productions, i.e., calculation of efficiency for prioritizing, ranking, and then simulation of efficiency for the factor investigation determination. To authenticate this amalgamation, 19 power plants [169], efficiency simulation was performed for 50 corporations [170], 102 bank branches [171], 45 countries [172], and 49 Indian business schools [173]. The classification of efficiency was also verified by studying 23 supplier companies [174], and 142 bank branches [175].

#### 3.3.5 Roadways safety examination with GIS

**Fig. 9:**
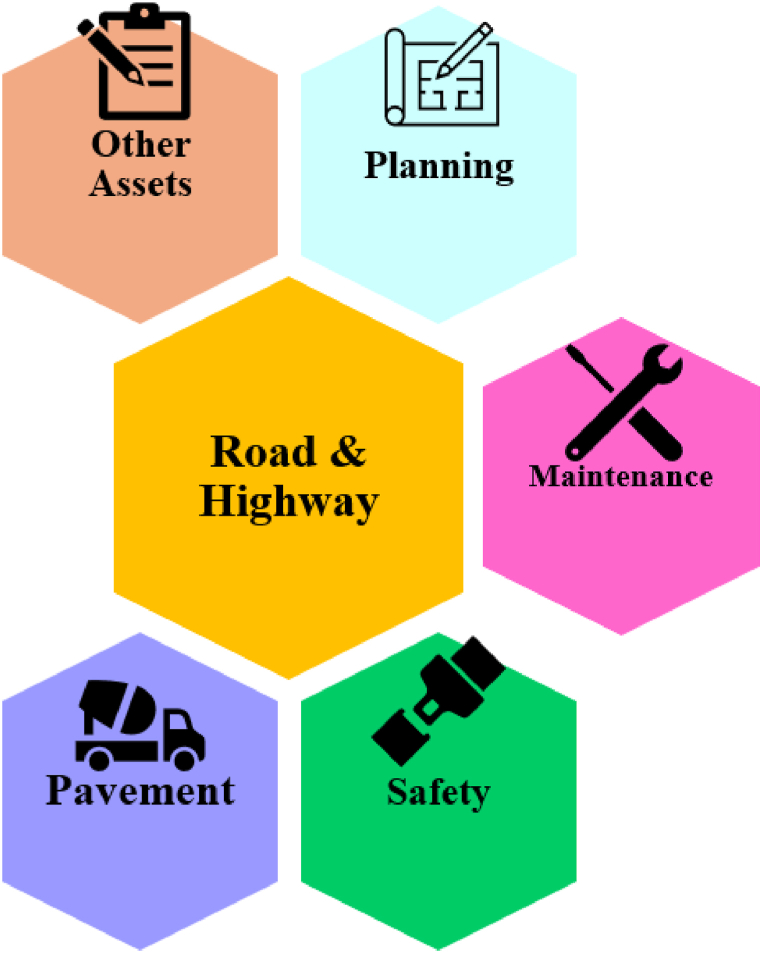
ArcGIS Road and Highway safety analysis (Source: https://www.esri.com/en-us/arcgis/products/arcgis-roads-highways/overview).

“Although geometrical perception might be augmented through the culture with unambiguous strategies comparable diagrams, before a relation of the unusual philological, beneath these erraticism dishonesties a communal set of geometrical perceptions. These perceptions countenance children and adults through minimal spatial language and no formal education, to catalogue geo-metrical procedures with to use geometrical correlations to characterize the neighbouring three-dimensional layout.” (Elizabeth S. Spelke-Harvard). The use of GIS methods has significantly enhanced the standing that provides a better understanding and decision-making process through the mediation of large datasets. The GIS that provided the graphics made it easier to classify collision extents that were next to the busiest roads in the most populated areas [176]. Throughout the roadways protection examination of the thoroughfare (M − 25), the GIS approaches provided appropriate datasets arranged the roadway traffic with accidents, and roadway physiognomies for the 70 sections [80]. The most dangerous segments arranged a possible cost of the crash basis used for the superhighways of Shanghai through a GIS application have been noticeably diagrammed [79]. The zonal crash frequency has correspondingly remained articulated complete the GIS, presenting connotation through the demographic, numerous social-economic, and transport organization influences [94]. GIS-based techniques were used in Belgium to model accident data on a local scale, allowing for the documenting of the many zones with incredibly high crash statistics (Fig. 9). It permitted the additional effective reorganization of the resources and additional efficiency of road safety) administration in Belgium [177].

#### 3.3.6 Methodology of ANN-GIS

The ANNs algorithms have been presented for example the map-based instrument to the GIS toward achieving an extrapolative competence for the combined procedures [178]. The GIS in amalgamation through the ANNs was more prevalent in the irrigation, Agriculture, geoscience fields, and meteorology and it has been confirmed in the road safety field through the application of deep learning procedure Recurrent Neural Network (RNN) to simulate some traffic crashes severity of injury on behalf of the North-South Expressway-Malaysia [93]. Beforehand that method had been used with the landslide susceptibility applying landslide manifestation influences fashioned through the assistance of the ANNs procedure [179], simulation of sediment in Gothenburg harbour [178], uncovering the flooded vulnerability in the River Atbara, Main Nile, White Nile, and Blue Nile [180], Armandia lance late, Cerithideopsilla cingulate, and macrobenthos habitat probable mapping concerning Macrophthalmus dilatates [102], simulation of tunneling enactment mandatory in the monotonous tunnel enterprise to performance and works in the terms of constancy in addition to the influence on neighbouring environment [181], and learning the development patterns in the particular region [182], where the map's production of deforestation regulates the correlations among numerous spatial variables and deforestation, for example, forest disintegration, slope, elevation, road vicinity and payouts, and the type of soil (Table 5).

**Table 5:** Different types of road safety analysis methods and published literature.

Approaches	Reference
ANN-GIS approach	[93,183]
DEA-ANNs method	[[170], [171], [172], [173]]
ANNs for road safety analysis	[4,166,167]
DEA for road safety analysis	[84,87,[162], [163], [164], [165]]
GIS for road safety analysis	[42,43,84,[88], [89], [90], [91], [92],^177^,^184^]

### 3.4 Section analysis

The investigation designates the point-based standard density inspection to control and the customary line-based density examination to calculate the line section length in the specific component of the area and the overall number of statistics opinions in a constituent of the share. The road network method for density examination, recurrently applied through the GIS software and particular machine learning (ML) approaches is the neighbourhood procedure. The maximum of the above-mentioned traffic accident examinations were focused on the accident point incidence [89,128,129,137]. In the transmission circumstance to the disparate weights to the changed accident influences, it's imaginable to examine the contented road density substantially. Clustering examination of the spatial topographies is assembled on the administration of the commands established on the association of the convinced spatial features, to obtain the physiognomies of the spatial dissemination is a necessity-have connected substance [130]. In addition, the application of site selection tools like weighted overlay, fuzzy overlay, random forest, and frequency ratio model is examined to determine the best location for a hospital, fire station, shopping mall, ATM, and market. Additionally, several researchers have used GIS techniques to study the site selection [[133], [134], [135], [136]]. The hotspot area documentation for the road accident disposed to zones has particular limitations due to the connection among lack of visualization, space and time, the priority of hotspot ranking, and traffic volume is not considered for this method [151]. A large area of accident-prone zone identification is more important to estimating the RTA database [185]. The hotspot compassion organization in Belgium to this weighting collection intended at the injury severity continued inspected through several investigators. In India, numerous megacities and cities used this weighting structure was efficaciously adapted to control accident-prone hotspots [83,[186], [187], [188]]. Road safety presentations are frequently pretentious through road conditions, population pressure, and vehicle speed limits, where consciousness is similarly an activating factor for road safety approximation [21,161]. Consequently, road safety inspection is supplementary important to investigative the risk assessment, where straight control was used for exposure estimation, also numerous consequences and multiple contributions are intended by altered preparation. The DEAs were applied on behalf of the possibility of equal valuation of various republics [22]. Detecting annual expansion in road safety was correspondingly attended through the DEA technique utilization [165]. The ANNs have continued to recurrently prompted in abundant science arenas for prediction determination [37]. In the safety investigation on the road, the ANNs were applied to inspect the crashes and finish the orientation to the roadway, driver, vehicle, and disorder attributes [166].

### 3.5 Future research direction and recommendation

Most of the GIS analysts, researchers, and planners used network analysis tools to investigate the fastest route, shortage distance, nearest store, and many other facilities. Some new approaches must be added in the network analysis applications like distance of the different searching locations with the lowest time, recent time of rail, flight, bus or vehicles, where the customer searching the location. Many technology companies like Google, MapmyIndia, and different applications use the network analysis tool for shortage distance estimation, nearest stores, locations, distances, and other relevant results. Therefore, road network analysis is a mostly used application nowadays. The DEA is a standard such as the tool of optimization through its context of the theory in the linear software design. The DEA is most standard through the reference to the mechanism of benchmarking for risk evaluation and efficiency [189]. The influential procedure, the ANN, has been combined through the DEA to block this opening. Lastly, through this extrapolative ANNs possible with the DEA optimization capability accomplishment corresponding features, a protuberant modeling option is intended [[189], [190], [191]]. The enactment of the DEA-ANNs procedure in the road safety ground on behalf of decision-making instruments on behalf of the presentation of the road safety examination was assessed. Training and related programs can also help the general people. Some new approaches like alarming vehicles for high speed or limitation numbers, training upon vehicle application, and sharing road accident death and injury databases to the commoners are more useful for accident reduction and awareness. Future research like reduction of individual vehicle uses, accident-prone areas high-security management like low speed, fewer vehicles in a particular time, and other related activities measurement approaches and hassle-free monitoring methods are more important for this purpose.

## 4 RS-GIS application in land use study

### 4.1 Different types of satellite data

**Fig. 10:**
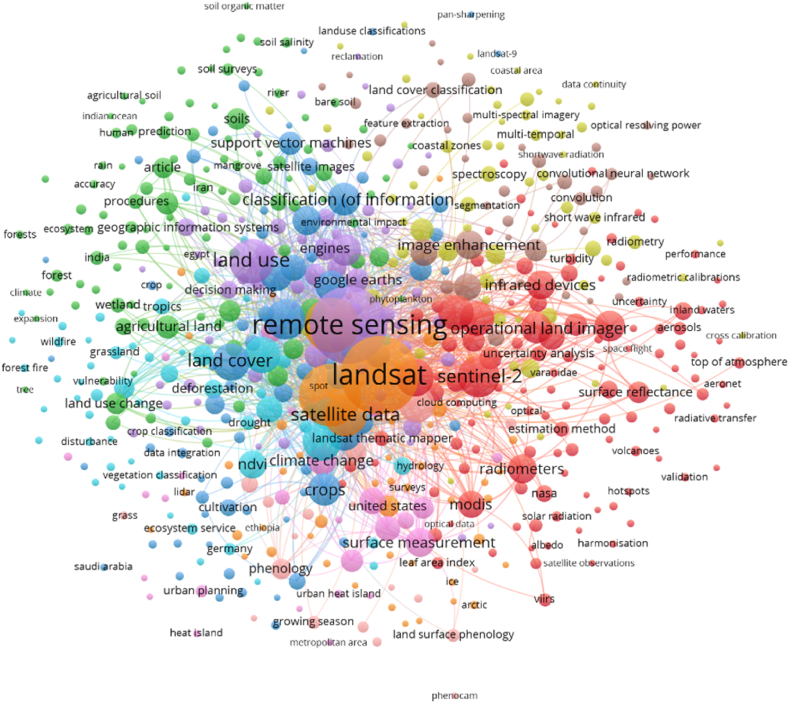
Different datasets and models applications in Land use study.

Different satellite datasets were applied for the land use studies because of different datasets resolution, areal conditions, and availability of the satellite images. Three types of satellite datasets were applied for LULC investigation multi-spectral, hyper-spectral, and microwave data. In the previous studies, multi-spectral datasets were widely applied like Landsat, LISS-III, and MODIS, but those imageries are moderate to low-resolution datasets, and micro studies are not properly investigated due to these datasets. Furthermore, Sentinel-2 multi-spectral datasets were widely applied for details investigation and analysis. Using keywords such as Landsat, Sentinel, MODIS, LISS, and satellite data, the Scopus database is searched to find the datasets utilized in the land-oriented research. This analysis indicates that the most used satellite data for Land monitoring is Landsat after that Sentinel 1/2 has been used recently with a high-resolution database (Fig. 10). This dataset is a 10-m spatial resolution; therefore, details studies are demarcated and analyzed for future prediction. Some applications of different satellite data were mentioned (Table 6).

**Table 6:** Different types of satellite datasets, data source applications, and literature.

Datasets/References	Instrument	Source of the Data	Advantage
Landsat 8 OLI/TIRS [15,37,125,[192], [193], [194], [195], [196], [197], [198], [199], [200]]	OLI/TIRS (Operational Land Imager/Thermal Infrared Sensor)	USGS (United States Geological Survey) https://earthexplorer.usgs.gov/	Helps on behalf of agriculture, wetlands, settlement, LULC, biomass estimation, shoreline change analysis, Vegetation, and land-related information
Landsat 7 ETM+ [197,201,202]	Enhance Thematic Mapper Plus	USGS https://earthexplorer.usgs.gov/	Helps on behalf of agriculture, wetlands, settlement, LULC, biomass estimation, shoreline change analysis, Vegetation, and land-related information
Landsat 4-5 TM [15,37,50,52,197,[203], [204], [205], [206], [207], [208], [209], [210]]	Thematic Mapper	USGS https://earthexplorer.usgs.gov/	Helps on behalf of agriculture, wetlands, settlement, LULC, biomass estimation, shoreline change analysis, Vegetation, and land-related information
Landsat 1 MSS [50,211]	Multispectral Scanner System	USGS https://earthexplorer.usgs.gov/	Helps on behalf of agriculture, wetlands, settlement, LULC, biomass estimation, shoreline change analysis, Vegetation, and land-related information
Sentinel- 2 [47,196,205,[212], [213], [214], [215]]	2A/ 2B	Sentinel Scientific Data Hub https://scihub.copernicus.eu/ USGS https://earthexplorer.usgs.gov/	Helps on behalf of agriculture, wetlands, settlement, LULC, biomass estimation, shoreline change analysis, Vegetation, and land-related information
Sentinel-1 [205,[216], [217], [218], [219], [220], [221]]	1A/ 1B	Sentinel Scientific Data Hub https://scihub.copernicus.eu/	Microwave datasets were applied for flood area detection, vegetation monitoring, crop identification, and precision agriculture
LISS-III [48,49,[222], [223], [224], [225]]	Linear Imagine Self Scanning System – III Resourcesat 1/Resourcesat 2	ISRO Bhuvan Indian Geo-Platform https://bhuvan.nrsc.gov.in/	Agricultural produce monitoring, forest mapping, water resource management, and urban/rural structure growth.
MODIS (Moderate Resolution Imaging Spectroradiometer) [36,[226], [227], [228], [229], [230], [231]]	NASA Terra Satellite	NASA MODIS data https://modis.gsfc.nasa.gov/data/ USGS https://earthexplorer.usgs.gov/	Air quality measurement, Land resource change analysis.
DEM (Digital Elevation Model) [52,216,232,233]	Advanced Thermal Emission and Reflection Radiometer (ASTER), Space Shuttle Radar Topography Mission (SRTM), Global Digital Elevation Model,	ISRO Bhuvan Indian Geo-Platform of https://bhuvan.nrsc.gov.in/ USGS https://earthexplorer.usgs.gov/	Estimation of a terrain elevation, aspect, and slope.

### 4.2 Satellite image classification techniques

Different satellite image arrangement methods were applied on behalf of a LULC categorization with the examination, therefore some most important classification methods are found in the previously published articles where the most applied classification method is supervised classification and new RF and SVM learning classifiers are also useful (Table 7).

**Table 7:** Different types of classification techniques and published literature.

Techniques	Reference
Supervised Classification	[11,15,53,69,70,73,98,110,112,113,116,223,234,235]
Support Vector Machine learning	[[236], [237], [238], [239]]
Random Forest Classifier	[221,[240], [241], [242]]
Gaussian maximum likelihood (GML) classifier	[111]

### 4.3 LULC prediction

#### 4.3.1 Markov chain

The LULC prediction models are widely applied for the earth's surface feature alteration prediction. Consequently, a Markov Chain algorithm was also applied on behalf of the LULC simulation using previous land classification maps. Furthermore, the algorithm of the Markov Chain is a random value where the LULC prediction probability intervals are dependent on the total number of values in the previous time. The algorithm of Markov Chain outputs was based on a probability of transition [114]. The LULC change predicted transition probability matrix was applied from one time to another time where the projection basis is used for the later periods. This model is widely applied for future LULC prediction and simulation over time using previous datasets [105,[243], [244], [245]]. Several applications are noticed in this model for landscape modeling and future prediction of the landscape [112,[246], [247], [248]]. This model predicts the future landscape based on the previous LULC features but it's not good enough for delineating the spatial landscape changing pattern, because this is not a model for spatial distributions of the landscape where projected change is authoritative happening sympathetic of a possible impression. An elementary Markov chain algorithm is like LULC by particular topic in the earth's forthcoming (t+1) might be indomitable by way of a current LULC purpose (t). equations (18), (19)) is used for Markov chain estimation.

(18) $$L_{\left(t+1\right)}=P_{ij}\times L_{\left(t\right)}$$

and,

(19) $$P_{ij}=\left[\begin{array}{ccc} P_{11} & P_{12}\ldots \text{.} & P_{1m}\\ P_{\begin{array}{c} 21\\ .\\ .\\ . \end{array}} & P_{22}\ldots & P_{\begin{array}{c} 2m\\ .\\ .\\ . \end{array}}\\ P_{m1} & P_{m2}\ldots \text{.} & P_{mm} \end{array}\right]$$

where the $L_{\left(t+1\right)}$ and $L_{\left(t\right)}$ were a LULC position at period (t+1) while (t) correspondingly. $0\leq P_{ij}<1$ with $\sum_{j+1}^{m}P_{ij}=1$, (i, j = 1, 2, …, m) is denote that a probability matrix of transition.

#### 4.3.2 Hybrid algorithm of cellular automata (CA)

The disconnected dynamic algorithm CA system was the condition of the cell time around (t+1), which is directed through the neighbouring cells in periods with the pre-define transition procedures applied [52,249]. The CA model predicts the spatial and temporal variation or evaluates the two-dimensional things [57,104]. The Local examination applied as the communal assessment amongst the altered situations of cells inside the neighbourhood allowing for the communication concentration of the cell that reductions through an intensification of a detachment among the cells, and the magnitude of a region incorporated in a neighbourhood [103,112,244,247]. The study outcome is a LULC modification of a fundamental pixel. A LULC opposition concentration expresses the central and neighbouring cells association.

The CA model demonstration discrete alteration classification contains the four elements, like the cell automaton neighbourhood demarcated as entirely cells tumbled within a radius everywhere the actual cell (ℵ), that is a separate framework or a physical atmosphere (L), a probable set states in the every $i^{th}$ lattice cell at the period phase t(ε), and a local transition rule is (δ). The CA model estimation by equation (20), (21), (22), (23), (24)) and the stepwise formulations are added below.

(20) $${ℵ}_{s}r=\left\{\frac{i_{1},i_{2}}{dist\;\left(i_{1},i_{1}\right)}\right\}<r$$

where $dist\;\left(i_{1},i_{1}\right)=\sqrt{{(i}_{1}^{2}+i_{2}^{2})}$, and ${ℵ}_{s}r$ indicates that the neighbours of the particular cell.

(21) $$\delta :\Sigma^{\left|ℵ\right|}\to \Sigma :U_{j\epsilon {ℵ}_{i}\left(t\right)}\sigma_{j}\left(t\right)\to \sigma_{i}\left(t+1\right)$$

where the calculation formal that complaint of a $i^{th}$ cell in the subsequent time stage (t+1) was calculated through the δ, constructed arranged the disorder of the neighbourhood cells.

(22) $$∁\left(t\right)=U_{j\varepsilon L}\sigma_{j}\left(t\right)$$

In the following stage, using a confined changeover instruction to altogether a cell happening the CA's framework, a subsequent conformation of a CA model might be calculated through this persuaded worldwide map.

(23) $$G:\Sigma^{L}\to \Sigma^{L}:∁\left(t\right)\to ∁\left(t+1\right)$$

However the CA model monitors the above-mentioned stages and mathematical calculations to monitor with calculate happening a procedure, the succinct and customary CA monitored finished Equation (24).

(24) $$S^{t+1}=f\left(S^{t},N\right)$$

where, the S denoted that every possible criterion of the CA model; f is represented by the change of the transition, which is stated from t to t+1; N is defined as the neighbourhood of every cell of the providing values of the function f.

#### 4.3.3 Land change modeler (LCM)

The LCM measures LULC variations among two different periods, S1 (previous classification) and S2 (final Classification). This modular calculates the variations and demonstrates the outcomes by applying the cartographic or statistical technique. Finally, this modular forecast of the future LULC of a particular area is based on the relative transition probable maps. The LCM modeling methods trust two different types of participation maps, (i) LULC maps at times previously the standardization period and (ii) explanatory variables maps. An algorithm of LCM is established by Ref. [250] by way of an empirically parameterized LULC modification prognosis instrument toward maintenance of the general planning activities range. Established on a historical LULC modification investigation, the arrangement progresses an experiential algorithm of a connotation among landform changeovers with an explanatory variable set. The forthcoming alteration mappings established continuously this experiential association with a quantity-derived prognostication since the Markov Chain algorithm. These outcomes were a business-as-usual (BAU) change prognostication deprived of independent interference [122,251].

#### 4.3.4 GEOMOD

The GEOMOD modular was applied for the accomplishment LULC alteration forecast. The GEOMOD algorithm was a grid-oriented LULC change procedure, which forecasted a LULC three-dimensional decoration alteration backward and/or forward in time [252,253]. The GEOMOD forecast the modification among two different land groupings. The applier has to source a map of commencement evidence regarding and time the grid-cells number of every classification at a conclusion period. On the suitability map, the GEOMOD categorizes cells interested in the classification [106,121,254]. The applier has to deliver only one commencement LULC map and the modification approximation. Correspondingly, the GEOMOD modular can generate automatically and empirically an appropriate map based on numerous carters and a LULC diagram since the particular point in the period [106,121,252].

#### 4.3.5 Modules for land use change evaluation (MOLUSCE)

A MOLUSCE plug-in, automatically cooperatively through an Asia Air Survey Co. Ltd. with the NEXTGIS on behalf of the QGIS 2.8.4 software, is applied toward the prediction of the LULC modification surrounded by a region. A boundary of the MOLUSCE model plug-in was informal toward apply with comprises the elementary components like input variables, region modification examination, changeover potential modeling, forecast, and validation [98]. The open-source podiums apply progressively accumulative in the private sectors and public, and one like method was the MOLUSCE algorithm calculated surrounded by QGIS software toward investigate the forecast of landform variations. The operator MOLUSCE algorithm boundary was accessible with comprises acknowledged procedures that might be functional happening the LULC variations examination while green space of urban, croplands environments [10,113]. The algorithm can formulate the probability matrix or changeover possible through a Markovian method with train forecast procedures established arranged different altered techniques named logistic regression (LR), artificial neural networks (ANNs), weights of evidence (WoE), and multi-criteria decision analysis (MCDA). The MOLUSCE uses a multi-layer perceptron through the sigmoid purpose of numpy.tanh. Consequently, target variables (transformation diagram classifications) would be surmounted to the (−1, 1) intermission throughout imitation coding in its place of a (0, 1), (NEXTGIS).

#### 4.3.6 ANN models

The LULC modification and forecast simulations were accompanied by the application of an ANN-Cellular Automata algorithm [[255], [256], [257]]. The ANNs are applied toward regulating a LULC changeover possibility by applying numerous production neurons aimed at forecasting numerous landform variations, surrounded by an ANN-CA construction [117,169,258]. The CA algorithm was applied toward modular landform variations by using transition probabilities after an ANNs knowledge procedure [259,260]. A complete investigative process was designated in subsequent periods, with QGIS and the MOLUSE plug-in segments applied on behalf of an ANN-CA demonstration. equations (25), (26)) is used for LULC classification based on ANN model.

(25) $$X={\left[x_{1},x_{2},x_{3},\ldots \ldots .x_{n}\right]}^{T}$$

where x_i_ indicates a $i^{th}$ characteristic, while T is representing the rearrangement.

After that, a demonstration of transition probability is done through the ANNs. Neural network arrangement contains 3 different deposits, specifically, a contribution, and output, with a hidden stratum. Every three-dimensional adjustable was accompanied through the neuron in a contribution level subsequently scaling surrounded by the assortment [0, 1]. Consequently, 17 neurons conforming toward 17 characteristics were applied in a contribution level, where a concealed level, a signal conventional through a $J^{th}$ neuron, ${n\text{et}}_{j}\left(k,t\right)$, since an input level aimed at the $k^{th}$ cell by period t is calculated through:

(26) $${n\text{et}}_{j}\left(k,t\right)=\sum_{i}\omega_{i,k}x_{i}^{\prime }\left(k,t\right)$$

#### 4.3.7 Algorithm of LR or (logistic regression)

McFadden established the logistic regression model, the multivariate analysis category model [261], which was future applied by Landis & Zhang to set up their UGM [262,263]. The Regression determines empirical associations among the binary dependent and independent categorical and unremitting variables [264,265]. The forecast dependent adjustable in the LR modular is a possibility function of the specific subject determination being in groupings; behalf of example, a changing LULC probability class established scores on set on forecaster variables like immediacy toward transportation strips, etc. [7,115,118,266]. The LR is calculated through 27 to 29.

(27) $$y=\left(b_{0}+b_{1}x_{1}+b_{2}x_{2}+\ldots \ldots \ldots \ldots +b_{n}X_{n}\right)$$

(28) $$y=\log\left(\frac{P}{1}-P\right)$$

(29) $$\log\left(\frac{P}{1}-P\right)=\left(b_{0}+b_{1}x_{1}+b_{2}x_{2}+\ldots \ldots \ldots \ldots +b_{n}X_{n}\right)$$

while, *x*_*1*_*, x*_*2*_*, … x*_*n*_ representing the dynamic influences, *y* indicates a linear amalgamation of explanatory variables purpose on behalf of the linear line relationship, b_0_ indicates interrupt of the algorithm, b_1_, b_2_ ….b_n_ were denoted that the algorithm limitations or regression coefficients while those might be predictable, P indicates that the alteration probability of the cell since rural, fringe to the urban. Purpose y is symbolized as the record of the likelihood ratio or odds that the reliance on mutable is 1 (Eq. (27).

#### 4.3.8 Algorithm of WoE

The WOE was the algorithm established continuously a Bayes instruction for conjoining datasets to forecast events occurrence [267]. The technique applied the prior (unconditional) probability concept and posterior (conditional) probability [268]. A comprehensive explanation of the technique is obtainable in the Bonham-Carter, individual an ephemeral explanation is provided in the book. If an area is separated into component cells (pixels) with an immovable scope, and the entire area is t, formerly $N\left\{T\right\}=\frac{t}{s}$ was an entire pixel's number happening in an investigated zone. Currently, uncertainty nearby is several pixels, N{D}, having an incidence D, then the prior probability of an incidence is deliberate as (equations (30), (31))):

(30) $$P\left\{D\right\}=\frac{N\left\{D\right\}}{N\left\{T\right\}}$$

If, undertaking a dualistic forecaster design B conquering N{B} pixels happen to happen a part with the known boreholes amount surrounded by this design, that is, P{D∩B}. A location favourability of the manifestation assumed an attendance of the forecaster design can be communicated through conditional probability.

(31) $$P\left\{D|B\right\}=\frac{P\{D\cap B\}}{P\{B\}}=P\left\{D\right\}\frac{P\left\{B|D\right\}}{P\{B\}}$$

where P{D|B} is denoted that the posterior probability of an incidence assumed the predictor pattern attendance, P{B|D} indicates that the posterior probability of existence in the forecaster decoration B, assumed an occurrence presence of D, P{D} is a forecaster arrangement of the prior probability.

#### 4.3.9 AHP-based multi-criteria evaluation (MCE)

This hybrid forecasting approach is another constituent which is the MCE of a landform modification with heavy influences [133,269]. Consequently, toward harvest changeover possible diagrams of an algorithm, MCE, AHP, and fuzzy membership purpose were useful [20,134,[269], [270], [271], [272]]. This AHP technique is used to regulate driving influence weights through a pairwise assessment. Thus, the LULC transition weighting potential is empowered by this AHP technique with orientation to potential diagram sets that include limitation impacts. A changeover probable diagrams designate the pixel aptitude toward modification since one category toward additional or continue unaffected.

(32) $$\mu =\cos^{2}\;\alpha \alpha -\left(x-point\;e\right)/\left(point\;b-point\;e\times \frac{pi}{2}\right)$$

(33) $$\mu =\frac{1}{{\left(1-\left(\frac{x-p^{2}}{p^{2}-p^{1}}\right)\right)}^{2}}$$

(34) $$L\left(x\right)=\left\{ \begin{array}{cc} 1 & if\;x\leq a\\ \frac{b-x}{b-a} & If\;a<x\leq b\\ 0 & if\;x>b \end{array}\right.$$

Three different equations denoted that the different calculation matrix like sigmoidal (eq. (32)); J-shaped (eq. (33)); and linear function (eq. (34)).

#### 4.3.10 Random forest model

The multi-decision tree ensemble classifier, RF classifier generates numerous pronouncement trees by applying the haphazard assortment of the exercise examples and the variability. In the current era, ensemble-based learning methodologies are commonly used in an RS method. This was presented through the author Breiman [273], which associations K binary organization with the regression trees (CART) by way of this non-parametric classifier, there are no statistical expectations that have to be complete before the dissemination of the datasets [196,221,237,241,274]. The formulation of RF model is 35 and 36.

(35) G(Xi)=∑Jj−1P(Xi−Lj)(1−P(Xi=Lj))=1−∑JjP(Xi−Lj)

(36) $$\text{OOB}\;\text{error}=\left(1-\frac{1}{N\sum i\varepsilon \text{oob}\delta i}\right)\times 100\%$$

where G(Xi) indicates that the impurity index, P(Xi−Lj) is denoted that the projected categories, and Xi=Lj is probabilities. N represents the number of observations and δi is precision indicator adjustable.

This estimate of mistake is recognized, for instance, in an out-of-bag (OOB) computation error. Without multiple prunings, each decision tree was individually constructed, and each node was divided using a user-defined features number (Mtry), which was chosen at random. Through increasing the forestry up to a user-defined number of trees (Ntree), the algorithm generates trees that have low bias and high variance [273]. To arrive at the final classification choice, the class assignment probabilities are discussed using fully constructed trees, and the arithmetic mean is utilized. This new unlabelled dataset input was subsequently estimated against all decision trees produced in the collaboration, with each tree voting for a membership class (Table 8). The RF was the most common ML algorithm applied for the regression study and classification [273].

**Table 8:** Different types of LULC prediction methods and published literature.

References	Prediction model use	Study area	Outputs
[112]	CA-Markov Chain Model	Allahabad district of Uttar Pradesh, India	Landsat 5 TM and ETM + use. Classification 1990, 2000, and 2010. Simulated years 2010 and 2020.
[113]	CA-Markov Chain Model	Dhaka mega-city, Bangladesh.	Landsat 5 TM use. Classification 1990, 2000, and 2011. Simulated years 2022 and 2033.
[98]	ANN Model	Gautam Budh Nagar, India	Landsat 5 TM and Landsat 8 OLI/TIRS use. Classification 2001, 2010, and 2016. Simulated years 2019, 2022 and 2031.
[114]	CA-Markov Chain Model	Karnataka, India	Landsat 5 MSS, TM and LISS-III use. Classification 1973, 1992, 199, and 2007. Simulated years 1999 and 2007.
[115]	CA-Markov Chain Model, Markov Chain, Logistic Regression	Lucknow, India	Landsat 5 TM, ETM+ and OLI use. Classification 1993, 2003, and 2013. Simulated years 1999 and 2007.
[116]	CA-Markov Chain Model, Logistic Regression	Phuket Island, Indonesia.	Landsat 5 TM, ETM+ and OLI use. Classification 1995, 2000, and 2014. Simulated years 2014 and 2026.
[117]	artificial-neural-network-based cellular automaton (ANN-CA)	North Sumatra	Landsat 5 TM and OLI use. Classification 1990, 2000, and 2010. Simulated years 2010, 2050, and 2070.
[103]	CA-ANN model, MOLUSCE tool (QGIS)	Nagpur City, Maharashtra, India	Landsat 5 TM, Landsat 7 ETM+ and Landsat 8 OLI/TIRS use. Classification 2000, 2005, 2010, 2015 and 2020. Simulated years 2025.
[118]	Markov chain, cellular automata, multi-criteria Evaluation	Hyrcanian region, Gilan, Iran	Landsat 5 TM, ETM+ and OLI use. Classification 1989, 2001, and 2013. Simulated years 2013, 2025, and 2037.
[52]	AHP automated cell model with the CA model	Qazvin City, Iran.	Landsat 5 TM and OLI use. Classification 1996, and 2016. Simulated years 2026.
[10]	CA-ANN model, MOLUSCE tool (QGIS)	Kolkata, India	Landsat 5 TM and OLI use. Classification 1990, 2000, 2010, and 2020. Simulated years 2030 and 2050.
[119]	Random Forest Classifier	Northwest Sheridan County.	SLC ETM + data use.
[120]	Frequency ratio, logistic regression, weight of evidence	Kendrapara, India.	Flooded area prediction using Sentinel-2, AVIRIS-NG, and Sentinel-1.
[253]	Logistic regression-Markov chain, Geomod model	Marand, Iran.	Landsat 5 TM and OLI use. Classification 1990, 2002, and 2014. Simulated years 2014 and 2025.
[106]	hybrid GEOMOD-Markov Chain model	Sufichay River catchment, Iran.	Landsat 5 TM and OLI use. Classification 1985, 2000, and 2015. Simulated years 2015 and 2030.
[121]	GEOMOD model	Kanakapura Region, India	Landsat 5 MSS, TM, and ETM + use. Classification 1973, 1992, and 2000. Simulated years 2000 and 2015.
[122]	Land Change Modeler (LCM)	Muzaffarpur city, India.	Landsat 5 MSS, TM, and use. Classification 1988, and 2010. Simulated years 2000 and 2015.
[123]	Land Change Modeler (LCM)	Ganga River Basin, India	Landsat 5 MSS, TM, and use. Classification 1985, 1995, and 2005. Simulated years 2030, 2060 and 2090.

### 4.4 Section analysis

Transportation development is triggering air pollution, which is one of the dominating factors for the development of new diseases in the human body. Lung cancer, asthma, respiratory infraction, and many other diseases are noticed due to air pollution. The unexpected expansion of the urban landscape impacts the natural environment and increases urban vulnerability. Vegetation degradation, insufficient water bodies, unsatisfactory groundwater zones, temperature variation, land subsidence, and waterlogging-related difficulties are increased due to urban expansion [275]. Urban landscapes are gradually populated and increasing scenarios of global climate change, urban areas are facing huge amounts of temperature variation, green space deficiency, which is triggering heat stress, high-rise building increases the land subsiding and increased soil erosion, precipitation fluctuation, oxygen deficiency, and high temperature [53,276,277]. Infrastructural development of the urban areas is increased transportation accessibility gradually triggered air pollution, that's why urbanized extents were mostly polluted somewhat in the rural-side and fringe regions. A Markov Chain algorithm productions were established on a transition probability [114]. The LULC alteration projected transition probability matrix was functional from one time to additional time where the projection basis is applied for the later time epochs. The CA model forecasts the temporal and spatial evaluation or variation of the two-dimensional things [57,104]. The CA model is used for the global LULC alteration examination with the assistance of simulation approaches. The GEOMOD prediction is the alteration between two different land surface consortiums. The user has to foundation a map of commencement evidence regarding and time the grid-cells number of every classification by the side of the assumption period. In the suitability map, the GEOMOD categorizes cells into one of the classifications [106,121,254]. The open source portion application is progressively materialistic in the private sectors and public, and one like technique was a MOLUSCE algorithm premeditated surrounded by the QGIS open source software to investigate with the simulation of the LULC variations [278].

Worldwide population pressure is the main dominating factor for urban expansion, food scarcity, deforestation, shortage of agricultural productivity, and many more. In cities and urban environments, land surface temperature is currently a major factor in heat stress. The temperature dissimilarity and heat stress-related studies are produced using remote sensing data from Earth observational space. Globally glaciers melting because of the temperature increase, worldwide sea-level rise (SLR), shoreline change, vegetation shortage, and population pressure are triggering factors for global climate change results. Adaptation, mitigation, and strategy building are the principal planning purposes for urbanized regions' sustainable development. Urbanization phenomenon is universal representativeness throughout the world [279]. This condition was frequently poisonous to natural-physical surroundings comparable to agricultural land, water bodies, vegetation cover, etc. The urban areas are growing due to population pressure, and local settlement development due to economic, social, and political services with a principal component of a settlement or concrete external [280]. The quick change of urbanization occurring in developing nations in worldwide has not individual triggered demographic changes and urban development, but also increased the unplanned, unexpected, and haphazard alteration of the rural-side areas and accelerated urbanized sustainability [281]. Those conditions influence the water bodies, agricultural land, and natural environment [282,283]. The main reasons for the development of urban expansion are anthropogenic activities, population pressure, and infrastructural development [284]. This section is more important to identify the variation of the earth surface change analysis using remote sensing datasets where different types of satellite datasets application, different types of satellite image available, and LULC prediction methods are added to identifying the variation of the earth surface change analysis.

### 4.5 Future research direction and recommendation

Space-based spatial data analyses have some difficulties due to the large area coverage like spatial resolution, heterogeneous land features, and availability of the satellite datasets. Previous studies indicate that the Landsat imageries are widely used for spatial data analysis and implementation purposes [202,205,259]. Cloud cover was a main concerning influence on behalf of space-based spatial investigation because optical datasets can't penetrate the cloud. So, cloud-free image availability is a must be needed for proper investigation. Real-time monitoring of the drought has some difficulties, actual temperature variation, vegetation dynamics analysis, the spatial distribution of precipitation, and space-based soil moisture analysis are more cost-effective and time-consuming purpose. As a result, true drought analysis is much more difficult, but space-based studies can aid in appropriate implementation and the development of creative adaptation techniques specific to that region.

## 5 Road and land use

The spatiotemporal diversity of the road and infrastructural development are gradually triggering the variability of urban development where transportation creates some difficulties for nature and the population [37,285]. Therefore road accidents, traffic congestion, air pollution, and related issues arise, which may be controlled through the LULC planning and appropriate management system for low accident-proof areas, healthy air for livelihood, and reducing global warming through the transportation systems [16,37,50]. In addition to the road density producing significant air pollution, other elements that induce the thermal variance include population pressure, deforestation, urban amenities, and LULC change [18,286]. Some novel approaches are applied for reducing transportation-related pollution like electric buses, high not fuel transportation uses, and awareness can also build proper situations to control the pollution in the earth's surface. Furthermore, LULC and road infrastructural studies are supplementary advantageous on behalf of the planning with management determination, therefore proper techniques and approaches were more useful for the LULC and the road-related investigation.

### 5.1 General description

Numerous approaches are applied for urban planning and management purposes; therefore, some popular uses approaches are analyzed and reviewed for this investigation. The most widely used urbanization demonstration platform in the USA was the ITLUP (EMPAL/DRAM), whereas the TRANUS and MEPLAN techniques will be fully applicable in South America and Europe (despite further partial presentation occurring in North America). The other three groups MUSSA, UrbanSim, and NYMTC-LUM are presently operational, or appropriate adjacent to existing functioning, in one or additional practical surroundings. Every correspondence is remarkable in that it comprehends an interesting and significant methodology for land market demonstration, integrating unambiguous management of values in LULC and development. Many other urban modeling approaches also occur. Remarkable illustrations included in the models of microsimulation [101,[287], [288], [289]]; land accounting-type frameworks [290], optimization models [291,292]; and other Japanese, European, and Australian models [287,[293], [294], [295], [296], [297], [298], [299]]. The optimizing approaches were valuable for reconnoitring whatever optimum urbanizing conformations influence looks comparable, nevertheless were commonly never of straight apply in an investigation of transport influences with LULC strategies arranged an existing development of the urbanized regions. Since landform secretarial procedures were widely used but seldom included land use transport communications, they are neither fully integrated into their enterprise nor fully absorbed. Lastly, numerous other approaches are already discussed for urban design.

#### 5.1.1 ITLUP (integrated transportation and land use package)

University of Pennsylvania scientist Stephen Putman, (Philadelphia, USA) first developed and applied the ITLUP framework. There are two main models were applied DRAM (Disaggregate Residential Allocation Model) and EMPAL (Employment Allocation Model). Those were applied in a Lowry unoriginal procedure [300] to apportion the families (frequently through the 4 earnings groupings, however, supplementary classifications are conceivable), transportable decorations (private and public approaches), and engagement (frequently through the four types, supplementary comprehensive Standard Industrial Classification (SIC) federations are conceivable). Employment forecasts, travel and population patterns, household compositions, and rates of activity are the primary inputs used in exogenous inquiry. Comprehensive certification of the approaches is provided by Putman, with advantageous instantaneous descriptions also obtainable [301,302]. The ITLUP (the two models like EMPAL and DRAM) is the furthermost extensively applied for the spatial apportionment framework in today's USA. A current amount designates completed a dozen energetic US presentations [303], though around forty standardizations in the USA with other parts must be accomplished transversely. Researchers proceedings that a significant improvement of EMPAL/DRAM is its foundation in commonly obtainable datasets like, connected toward population, households, and employment) [302]. Conversely, this was correspondingly renowned that this reproduces the methodology weakness; specifically, those approaches don't interpret aimed at land-based marketplace reimbursement developments. METROPILUS, a recent extension, aims to educate connections using GIS databanks by examining some methods close to the superior arrangement modularity. Those are the functions inside the ArcView explosive, while provisions linkages through the GIS database software ArcView, which was Windows companionable [303].

#### 5.1.2 Framework of the MEPLAN

An additional backdrop the proprietary software that governs MEPLAN is created by Marcial Technique and Partners Ltd., a secluded accessing corporation based in the United Kingdom. This was attractions continuously around involvement 25 years in applied combined urban modeling, with exertion proceeding package of themself software commencement happening the year of 1985. It has remained applied to over 25 counties throughout the world, counting California, and Sacramento, with the US Cross-Cascades Corridor in. Comprehensive certification can be originated in the various previous investigation [[304], [305], [306], [307], [308]]. The MEPLAN is a collective approach: space is alienated into the economic activities, zones, households, and quantities (which are called ‘sectors’ or ‘factors’) are apportioned toward this region, with interactions movements amongst these influences happening the altered regions contribute increase toward the transport flows demand. In this outcome, communications between regions bounce an increase toward a request aimed at the transportable. The temporal alteration was forecast by allowing for consecutive opinions happening during the period. The land and floor area were “non-transportable,” meaning that money had to be spent in one place while those were being made. Values on behalf of other sectors' productions were recognized endogenously consecutively backbone sideways production manacles ingesting. The difficulties of travel ascending aimed at the assumed argument in the interval were apportioned toward the multi-modal system applying legit occupations on behalf of route choice and mode, captivating congestion account. Comments on transportation impairments that are relevant in the future, about the periods that occur in response to the transportation circumstances. The exogenous demand, which corresponds toward a ‘Lowry’ [309] undeveloped subdivision delivers a preliminary motivation on behalf of financial movement.

#### 5.1.3 MUSSA

MUSSA framework was first established by Professor Francisco Martínez for Santiago, Chile, which is the functioning model-based approach of the urbanized LULC with markets of the floor space. This was ‘completely connected’ through the systematic of the four different phases algorithm-based approach (recognized for example ESTRAUS); composed, collective approaches were mentioned such as the 5-LUT and deliver equilibrated LULC predictions with transportable request aimed at the Santiago. The algorithmic method would be used to look at many transports and/or LULC techniques, often tying transportation together through the core policy element. Martínez was providing the approach certification [[310], [311], [312], [313]]. The Buyer's challenge to exploiting this remaining (WP a smaller amount essentially funded), although wholesalers' effort toward exploits the price compensated. The stock of building was completed through designers consequently by way of exploit proceeds, assumed an ostensible ultimatum. The stock of building values was endogenously resolute surrounded by an equilibration procedure. Resolves on behalf of the stationary symmetry in a simulated time through regulating a building stock amount complete, the source answer, with customers' anticipation heights (predictable usefulness toward remain gained since their covering), the request comeback, until supply and demand balance. Usages the analysis of the traffic neighbourhoods by way of the situation three-dimensional component of the investigation, thus if the comparatively spatial disaggregation fine level. Furthermore, postponements toward additional spatial examination micro echelons were existence examined [311].

#### 5.1.4 NYMTC-LUM framework

Professor Alex Anas established the framework on behalf of the New York Metropolitan Transit Commission (MTC), New York, USA [314]. This was the shortened variety METROPOLIS, with the greatest current of the LULC sequence with accommodation marketplace approaches established by Anas concluded in the last double periods [[314], [315], [316]]. The unfailingly founded all the way through on micro-financial philosophy. The instantaneously approaches communication amongst labour, inhabited housing, and non-work portable marketplaces, commercial floor space, through unambiguous request demonstrations with source procedures happening in every circumstance. Prices for housing and floor space, as well as wages for workers and rent, were entirely endogenously indomitable inside an approach and utilized to arbitrate between supply and ultimatum developments within their relevant marketplaces. In its present state of implementation, the approach does not comprise abundant disaggregation of its foremost communication components (households, employment, and buildings). This is the present enactment, a LULC constituent was not combined through the request algorithm of travel. This has something to do with a contemporary MTC trip request algorithm in reception relationships, as the MTC technique is an outstanding procedure, and this is somewhat “connected” to it. Those were comparable to a circumstance aimed at the UrbanSim MUSSA, and DRAM/EMPAL.

#### 5.1.5 UrbanSim

The framework developed by Professor Paul Waddell serves as the operational method for the urbanized LULC with floor-space markets in the United States of Hawaii, Oregon, and Utah. An example might be accomplished in Eugene-Springfield, Oregon. Approaches are premeditated toward exertion in combination through the outmoded 4-step algorithm in the Eugene-Springfield, which is existence associated with the novel activity-oriented transportable approach in Honolulu, Hawaii. Though a preliminary expansion of an approach is commenced complete a consulting firm of Urban Analytics, further support and development of the approach is being done at the University of Washington. This approach with framework might be positioned happening a community province through an Oregon Department of Transportation, and the University of Washington will support its release and broadcasting complete Internet aspect by way of part of the NCHRP Project 8-32 (3), ‘Integration of Landuse Planning and Multimodal Transportation Planning’ [[317], [318], [319]]. Admittance to certification and a methodology (www.urbansim.org).

Approaches conclusion government was footpath reliant on necessitates the explanation on behalf of every transitional era. Request adjacent of an approach applies transportation zones examination by way of this three-dimensional examination unit (271 zones in the Eugene-Springfield application, 761 in Honolulu, over 1000 in Salt Lake City, Utah), thus provided that the appropriate acceptable spatial disaggregation level comparative toward numerous additional contemporary approaches. Happening a quantity adjacent, an approach applies a separate LULC allotment by way of a land development component with improvement, and manufacture those are individual approaches to date to apply the parcel as the important investigation unit. The approaches were extremely disaggregated compared to the greatest additional presently operational approaches. The Eugene-Springfield application has domiciliary categories that might be run applying observed households of great prejudiced example (and their accompanying comprehensive characteristics) happening fundamentally in a stationary microsimulation format. The approaches are based on the investigation of policy circumstances that comprise complete landform strategies, development organization principles such as the urban growth boundaries, mixed-use development, minimum and thoroughgoing concentrations, environmental restrictions improvement, happening expansion, with expansion estimating guidelines, in addition to a transportation infrastructure assortment and assessing guidelines touched through the accompanying transportable request approaches.

#### 5.1.6 The framework of TRANUS

This compendium was registered software established through the Modelistica in Venezuela, a sequestered stable route through Dr Tomas de la Barra. This is essentially an analogous manner of showcasing MEPLAN's engagement through the foundational principles of a primary approach handbook that was written in the early 1980s. An important article of the TRANUS was an application of the slightly additional constrained purposeful modeling set options and forms within the background permitting an additional set approach to model expansion comparative toward the MEPLAN. This is practical for the region's number in South and Central America and Europe. The approached TRANUS is a Maryland, Sacramento, and Baltimore, USA, districts and Oregon is accomplished, or this was underneath expansion. Aimed at comprehensive certification, some previous research has also applied those techniques [[320], [321], [322]].

### 5.2 Section analysis

Urban development and the density of settlement are increasing the temperature variation and increasing temperature influences urban vulnerability. The maximum important ecological inspirations were an urbanized land surface temperature (LST) alteration with distinctive thermal variation, this affected the urbanization interior micro climatology, anthropogenic heat emancipation, superficial dynamism alteration, construction energy ingesting, human thermal comfort, and atmospheric pollution [10,[323], [324], [325]]. LULC alteration examination was applied by numerous investigators [125,275,[326], [327], [328], [329]] and land surface temperature [37,230,330] was applied aimed at defining ecological diversification and the climatic alteration that generates worldwide problems. In the past periods, suburbanization in common and manufacturing urbanization, in individuals occurred as a major heavy demographic force and economic, social, and environmental alteration in India [331]. Recent urbanization and high human density have altered the overall methodological and environmental circumstances of the area, resulting in climate change. This condition increased the unsustainable development of the urban area, increased thermal variation, and an urban heat islands (UHI) formation. The improvement of settlement parts was reducing natural landforms covering similar water bodies, vegetation, and cropland, which increased an LST over the study area. The majority of the influencing factors were UHI, indicating that urban areas are often more temperate than rural ones. The UHI has influenced indigenous climatic conditions and undesirable influences on the populations. The inadequate green area increased the UHI and affected the local ecological condition. Urban parts might be eminent by their alteration in empirical ambient atmospheric temperature since neighbouring country areas are known as UHI decision [275,332].

Several urban modeling methods are also used for road urban analysis. Extraordinary photographs comprise the representations of the microsimulation [101,[287], [288], [289]]; land accounting-type frameworks [290], optimization models [291,292]; and additional Japanese, European, and Australian representations [287,[293], [294], [295], [296], [297], [298], [299]]. The trip generation and dissemination were recognized surrounded by the DRAM, immediately through the domestic position. Contrariwise, the EMPAL, and the DRAM recurrently are used independently with have been complementary in authentic submissions with other commercial travel application approximating methods. Technical constants on behalf of an ingesting of the space were changeable with approbation to the price, and prices for the space the protection application connections with the supply in each neighbourhood are documented endogenously by way of measure of a symmetry explanation recognized for every point in time measured. On the Earth, the forest or vegetation environment delivers a widespread opportunity range to anthropological life [209] nonetheless devastating population development, pollution, and rapid urbanization, can impair green spaces or vegetation environment by discontinuing a financial expansion [196]. Green space auditoriums an enthusiastic character in the worldwide climatic modification and speedy climate change can distress a photosynthetic procedure. Increased plant species-related issues primarily affect metropolitan areas. Scenarios of climate change have an impact on human habitat as well, and the earth's surface is marked by urban vulnerability in many places. Temperature fluctuations and shifting precipitation patterns further exacerbate issues connected to biodiversity. The alien species also changed due to climate change scenarios [333] and the spread of disease and pests also increased in recent times [31].

### 5.3 Future research direction and recommendation

Forthcoming associated investigations will be satisfied through an agent-based modeling procedure in an investigational region to associate an urbanization outcome and a landform diminuendos tendency. Furthermore, additional dynamic influences will be combined in the modeling procedure for example per capita income, inhabitants' dynamics, landforms market ultimatum, and the number of decorations. The seriousness of an accident near was also noteworthy in the evaluation of accident-prone region hotspots, in addition to the event itself, as it helped identify a coincidence via several impairments. Safety measurement is essential for reducing death cases, injuries, health issues, and speed-related activities because many accidents happen in high-speed vehicle transport. Therefore, novel adaptation strategies can also help in reduction of the road accidents. Future research can also help to prepare some percussion to protect drivers’ health and reduction of a road accident, like quick access to app-based shortage distance, speed control devices, awareness, restriction of commercial vehicles in the office or other essential times, used public vehicles for traveling and many more essential phenomena to reduce the road accident and increase road safety (Table 9).

**Table 9:** Different types of roads and LULC methods and published literature.

References	Model Use	Application
[334]	TRANUS model	Transportation-related economic development
[335]	TRANUS model	Transit-oriented development
[336]	TRANUS model	Land use and transportation-related study
[337]	TRANUS model	Roadways estimating on the landforms under altered expansion situations
[56]	TRANUS model	Urban growth model using various criteria
[338]	TRANUS model	Impact of LULC on Urban Mobility Patterns
[339]	TRANUS model	Forecasting of megacity growth.
[340]	TRANUS model	Combined Land Use and Transport Algorithm
[318]	UrbanSim	Organization of the metropolitan landform's usage and transportation preparation
[319]	UrbanSim	Transportation, Land Use, and Environmental Planning
[341]	UrbanSim	Public discussion and decision-making
[342]	UrbanSim	Integrated Land Use and Transport Model
[343]	NYMTC-LUM	Simulate traffic loads for roadway corrosion curvatures
[344]	NYMTC-LUM	Integrated Land Use planning
[345]	NYMTC-LUM	Forecasting with Dynamic Microsimulation
[346]	NYMTC-LUM	Integrated land use, environment, and transportation, modeling
[347]	MUSSA Model	Land use modeling
[348]	MUSSA Model	Land use equilibrium model
[349]	MUSSA Model	Integrated Land Use and Transport Model
[[350], [351], [352], [353]]	MEPLAN Model	Integrated land use and transportation model
[354]	ITLUP Model	Integrated land use and transportation model
[355]	ITLUP Model, UrbanSim Model	Integrated land use and transportation model
[[356], [357], [358]]	ITLUP Model	Integrated land use and transportation model

## 6 Investigational funding

Research financing is increasingly crucial for supporting hustle-free research as well as open-access publications. Therefore, many funding agencies have funded research or a particular project. Open-access publication is also better for the reader because this publication method increases high accessibility and public notice (Table 10).

**Table 10:** Funding information and published literature.

Funding	Non-Funding
[69,70,87,90,94,111,119,[359], [360], [361], [362]]	[1,6,10,21,32,52,[83], [84], [85],^88^,^89^,[91], [92], [93],^98^,^103^,^110^,[113], [114], [115], [116],^118^,[121], [122], [123]]

## 7 Discussion

**Fig. 11:**
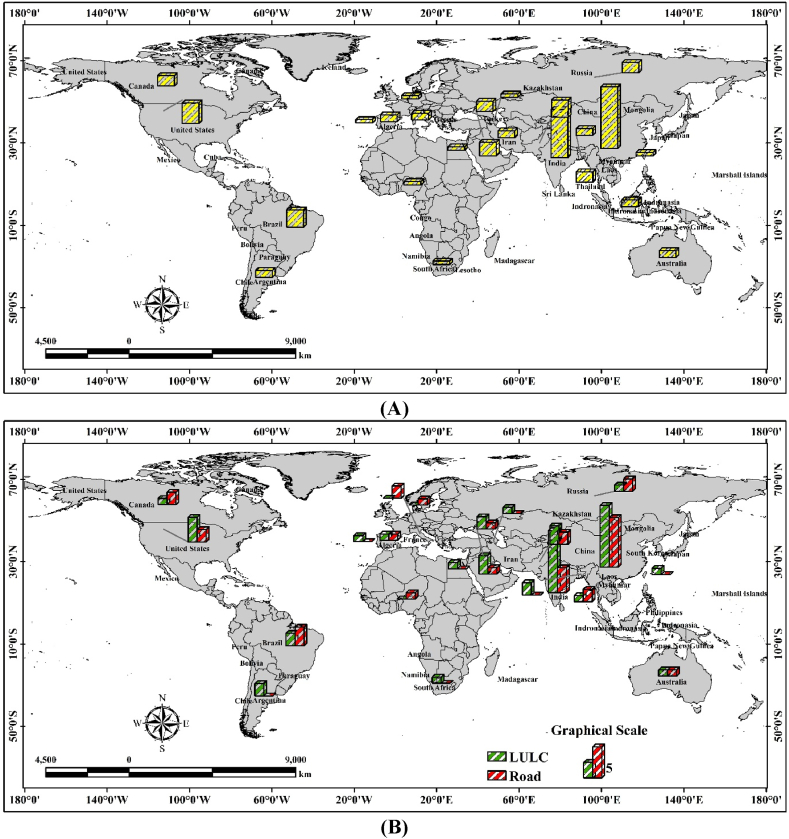
(A) Country-wise LULC and road applications (Based on the Scopus database), (B) Country-wise Road and urbanization-related study (Based on the Scopus database).

Earth's surface is gradually affected by anthropogenic activities and related issues like population pressure, residential area expansion, an increase in urban amenities, road network increase, vegetation damage, a decrease of open space, high land values for urbanization, and public health-related issues like industrialization, air pollution, transportation development, noise pollution, health-related issues like asthma, skin disease, and lung cancer are progressively intensification because of the high urbanization, and inhabitants growth, therefore the proper application and approaches are helpful to reducing those phenomena. Additionally, new methods and study studies might aid in lessening global transformation. Several apps were also released to minimize landform change and related problems. The road network is applied for the product and/or goods facilities from one plane to the additional habitation and this organization has more financial standards [27]. Around 75 % of cargo and/or people are shipped to the other regions of Sub-Saharan Africa [28]. In this district, the construction of roads and related infrastructure is approached with a 50 % charge for cities, road network growth, physical development, and/or potential social-economic [29,30]. Ghana and Kenya have particular road network-associated approaches which are increasingly growth with the spatial and social heterogeneity in the peri-urban zones [31]. Additionally, road traffic accidents (RTA) are the furthermost regarding influence in the contemporary years where urbanization intensification but the appropriate arrangement of the road network is extinguishing the planning and increasing the road accidents [32] (Fig. 11a).

The ecological phenomenon and biodiversity are progressively affected due to urbanization-related thermal variation, LULC change, forest health diversity, and other environmental and ecological characteristics. Consequently, the reasons and dissimilarity inspections of those circumstances are additional operative investigations that can assist in responsiveness to defend our surroundings, a healthier environment, and novel methodologies for monitoring those problems. The urbanized ecological eminence's most significant feature was the quantity of man-made landforms. A vast portion of the area is being used for a variety of purposes, including industrial, commercial, residential, and transportation. The physical possessions of the land planes, like material heat capacity, conductivity, soil moisture, and surface reflectivity, with the emissivity. A smart environment is a constituent of the SDGs that reduce below a sunshade of an environmental fortification. The growing threat of climate change and the depletion of natural reserves are currently the main issues. The physical, mental, and social well-being of individuals is significantly impacted by their surroundings [363]. The investigation is further significant to recognize the land modification analysis where human happenings with earth's crust variation are manipulating aspects for the incensement of the urban design, planning, and the management strategies like smart city, those analysis outcomes also designate the backgrounds of the land alteration and environmental characteristic where urban design, preparation, and management are essential for the invention of the smart city and the environmental fortification [[364], [365], [366]]. Worldwide road accidents and related problems were documented approximately 50 million of the grievance and around 1.3 million passing missing because of the RTA and associated happenings. It is very necessary to target the audience when and where the accidents are fashionable in order to reverse the downward trend in traffic accidents and/or related incidents. The large volume of material about accidents is also important for examining the specific complaint [33].

**Fig. 12:**
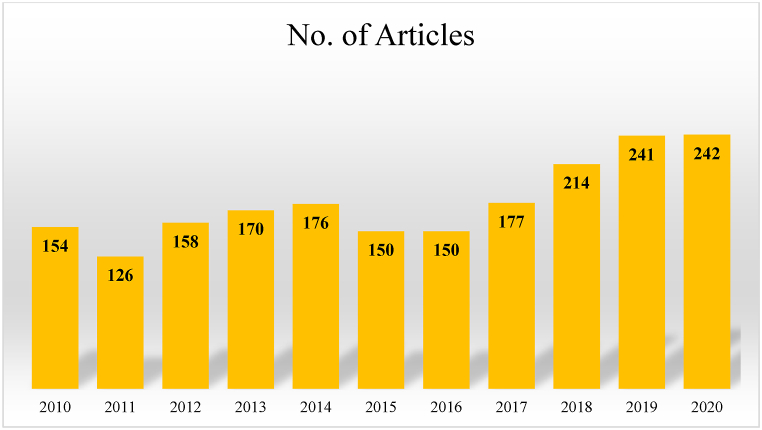
Published article in the domain of road safety and land use-related applications in different years.

**Fig. 13:**
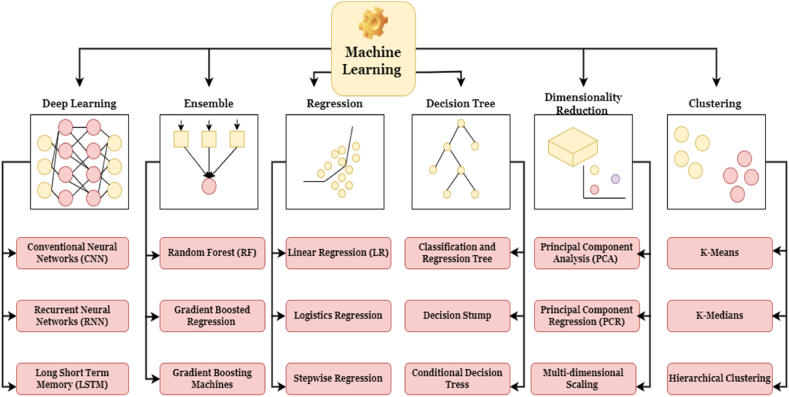
Machine learning applications for road and land use-related analysis.

In the numerous regions of the earth's surface, this review study employs GIS and statistical methods to analyse two distinct relevant components of the most significant feature, such as the road network and land modification research. Road safety is more essential aspect due to the high traffic jams, accidents in the linked road area, and high-speed vehicles used, no vehicle's speed and density area identification is gradually increased using GIS technologies, therefore shortage distance, point-to-point area analysis, density analysis applying kernel density approach and different methods are more useful (Fig. 11b). An additional component is the examination of LULC-related research, including the utilization of satellite information, classification techniques, and various methodologies for prediction. In the review articles, most of the investigations applied Landsat datasets for image classification, and some articles applied Sentinel-2 multi-spectral data. Mostly applied classification techniques are supervised, RM, SVM, and some are object-oriented classification methods. The most applied prediction model is MOLUSCE, a QGIS plug-in for predicting a future LULC using the previous two LULC datasets. Some recent articles have also applied the Markov chain, random forest, artificial neural network, and cellular automata for LULC simulation. Shortage distance and point-to-point analysis are more important for travellers who don't know the actual location and want to go to a particular location, therefore shortage distance can also help those people. Some cab services are also applied to this method for first and jam-free driving using various app-based technologies like Uber, Ola, Lyft, Cabify, Curb, Careem, Taxi EU, G7, Lecab, Taxi Amin, and Gett. Google map, MapmyIndia, mapsforeurope, and many others are most useful for maps using GIS techniques and mathematical algorithms for road and land information. In the review analysis entire road and land use-related analysis is gradually increased because of the high urbanization, landform change, and traffic congestion. Fig. 12 indicates the published literature in the study years. The literature review indicates some machine learning (ML) applications are applied for road safety and land use-related analysis which is mentioned in the road and land use literature and Fig. 13. Despite this, it is difficult to determine how the economy affects these research advancements by looking at the peer-reviewed articles because the relative numbers of research-based investigative articles in the two most prominent collections of decrease approaches are similar. It can be argued that modern research continuously examines the distinctive inclinations or positioning of the current investigation and may be secure in relationships of the outcomes due to the significant shift in the number of published scientific articles on specific extenuation procedures.

## 8 Conclusion

The conclusion of the current survey can be summarized as follows:

• The greatest number of critical difficulties that transportation engineers and manufacturers may find subjective. Suggested low-cost safety explanations and remedies might come from selecting an underwriting characteristic with a variable vehicle speed boundary and movement limitation on behalf of the unsafe pieces. Another part, a changing infrastructural, adjustment in the vertical and horizontal curvature can cost considerably more cost for traveling. Conversely, this arrangement can also assist with manipulative better resolutions as no one would favour to alteration of the construction of the complete state or national highways (like up to 110 km extended highways), consequently, choosing a maximum challenging fragment with a disentangling the security problematic of individual individuals' sections might correspondingly deliver decision-making lowest cost consequences. Additionally, relating the ANNs through the GIS-based technologies in road safety, related investigation, and LULC analysis arrangement can incorporate the functionality of ANNs, at a similar period, intensification possible applications set of the GIS approaches. RS and GIS approaches are appropriate for encouraging management tools of natural and environmental resources on an enormous temporal and spatial scale. In the investigation, satellite-based datasets landform diagrams were applied happening connotation through the socioeconomic and biophysical datasets to approximation the past and present fluctuations, with the forecast of forthcoming opportunities.
• Road safety measurement, shortage distance analysis, traffic congestion measurement, network analysis, and road-related issues identification are essential for infrastructural development and reducing those problems. Therefore, cost-effective services and adaptation policies are more useful. GIS and some statistical modeling can also help researchers, planners, and scientists with low cost, less time, and large area coverage.
• The modernizations of the past LULC are frequently complicated with incomplete characteristics of the spatial-temporal varieties. This is to a particular magnitude indomitable through the approaches within some discipline. For illustration, archaeology can stretch appropriate careful spatial information around LULC but frequently requires time resolution.
• Normally, well-structured consequence established on the LULC forecast assists in conducting urban growth in an additional maintainable method. Consequently, the multi-criteria AHP and hybrid CA-Markov procedure of a megacity's constituency landform alterations, simulation with an excellent of situations were obliged to monitor the city and the neighbourhood's position through manufacture suitable pronouncement on an urban cities area landform with ecological alteration throughout Worldwide in specific comparable USA, Europe, and Asian countries urban scheme determined toward observer their ecological possibilities with urbanization.
• This assists administrators, researchers, and planners with roadway safety activities toward identifying a crash of highest risk positions by a disaggregated equal through the precise crash physiognomies, i.e., crash type, cause, or the severity of the crash. Such a comprehensive examination might correspondingly deliver indispensable management to policymakers for healthier planning, management, and adaptation approaches, and well-organized application of the suitable possessions to the improvement of the regional level safety and percussion.

## CRediT authorship contribution statement

**Khalid Hardan Mhana:** Writing – review & editing, Writing – original draft, Visualization, Validation, Resources, Methodology, Investigation, Formal analysis, Data curation, Conceptualization. **Shuhairy Bin Norhisham:** Writing – review & editing, Writing – original draft, Visualization, Validation, Supervision, Methodology, Investigation, Formal analysis, Data curation, Conceptualization. **Herda Yati Katman:** Writing – review & editing, Writing – original draft, Visualization, Validation, Supervision, Resources, Project administration, Methodology, Investigation, Formal analysis, Data curation, Conceptualization. **Zaher Mundher Yaseen:** Writing – review & editing, Writing – original draft, Visualization, Validation, Supervision, Resources, Project administration, Methodology, Investigation, Formal analysis, Data curation, Conceptualization.

## Consent to publish

The research is scientifically consent to be published.

## Ethical approval

The manuscript is conducted in the ethical manner advised by the targeted journal.

## Availability of data and materials

Data will be supplied upon request from corresponding author.

## Funding

The research received no funds.

## Declaration of competing interest

The authors declare that they have no known competing financial interests or personal relationships that could have appeared to influence the work reported in this paper.
